# Genetic Dissection of the Regulatory Network Associated with High c-di-GMP Levels in *Pseudomonas putida* KT2440

**DOI:** 10.3389/fmicb.2016.01093

**Published:** 2016-07-20

**Authors:** María Isabel Ramos-González, María L. Travieso, María I. Soriano, Miguel A. Matilla, Óscar Huertas-Rosales, Laura Barrientos-Moreno, Víctor G. Tagua, Manuel Espinosa-Urgel

**Affiliations:** Department of Environmental Protection, Estación Experimental del Zaidín, Consejo Superior de Investigaciones CientíficasGranada, Spain

**Keywords:** *Pseudomonas*, biofilm, c-di-GMP, crinkle morphology, two-component regulatory system

## Abstract

Most bacteria grow in nature forming multicellular structures named biofilms. The bacterial second messenger cyclic diguanosine monophosphate (c-di-GMP) is a key player in the regulation of the transition from planktonic to sessile lifestyles and this regulation is crucial in the development of biofilms. In *Pseudomonas putida* KT2440, Rup4959, a multidomain response regulator with diguanylate cyclase activity, when overexpressed causes an increment in the intracellular levels of c-di-GMP that gives rise to a pleiotropic phenotype consisting of increased biofilm formation and crinkly colony morphology. In a broad genomic screen we have isolated mutant derivatives that lose the crinkly morphology, designed as cfc (crinkle free colony). A total of 19 different genes have been identified as being related with the emergence of the cfc phenotype either because the expression or functionality of Rup4959 is compromised, or due to a lack of transduction of the c-di-GMP signal to downstream elements involved in the acquisition of the phenotype. Discernment between these possibilities was investigated by using a c-di-GMP biosensor and by HPLC-MS quantification of the second messenger. Interestingly five of the identified genes encode proteins with AAA+ ATPase domain. Among the bacterial determinants found in this screen are the global transcriptional regulators GacA, AlgU and FleQ and two enzymes involved in the arginine biosynthesis pathway. We present evidences that this pathway seems to be an important element to both the availability of the free pool of the second messenger c-di-GMP and to its further transduction as a signal for biosynthesis of biopolimers. In addition we have identified an uncharacterized hybrid sensor histidine kinase whose phosphoaceptor conserved histidine residue has been shown in this work to be required for *in vivo* activation of the orphan response regulator Rup4959, which suggests these two elements constitute a two-component phosphorelay system.

## Introduction

In recent years cyclic diguanosine monophosphate (c-di-GMP) has emerged as an important second messenger in prokaryotic cells, where its role in defining the bacterial mode of life has been established. Changes in the levels of this signaling molecule are associated with phenotypic modifications related to virulence, motility, production of exopolysaccharides (EPSs), colony morphology, and expression of functions involved in biofilm development (Hengge, 2009; Römling and Simm, 2009). High intracellular levels of c-di-GMP favor the settlement of bacteria in the form of sessile populations (surface-attached cells and biofilms), whilst low levels of this molecule promote motility and the planktonic lifestyle. Thus, c-di-GMP is considered one of the key elements controlling biofilm formation and dispersal (Boyd and O’Toole, 2012).

The turnover of this second messenger is carried out by proteins containing the domains GGDEF or EAL/HD-GYP, which are responsible for the synthesis and hydrolysis of c-di-GMP through their diguanylate cyclase (DGC) and phosphodiesterase activities (PDE), respectively. Proteins containing one or both of these domains have been described in many bacteria. Often multiple of these proteins are found in the same organism and therefore there appears to be some redundancy (Römling et al., 2005; Ulrich and Zhulin, 2010).

The GGDEF/EAL containing protein Rup4959 was previously identified in *Pseudomonas putida* KT2440 by transcriptomic analysis of genes preferentially expressed in the rhizosphere of corn plants. This plant-beneficial bacterium colonizes the root surface and surrounding soil area (the rhizosphere) of different plants, a process that is accompanied by a global reprogramming of gene expression (Matilla et al., 2007). “*In silico*” analysis of Rup4959 showed that this protein is a multidomain response regulator with a REC-PAS-GGDEF-EAL architecture, similar to FimX of *P. aeruginosa* (Huang et al., 2003). Unlike FimX, which exhibits PDE activity (Kazmierczak et al., 2006), Rup4959 has been shown to present DGC activity (Matilla et al., 2011). REC domains are commonly found in response regulators of two-component phosphorelay signal transduction systems, which typically allow bacteria to regulate cellular processes in response to environmental signals (Galperin, 2006). On the other hand, PAS domains are normally associated with signaling proteins and act as sensors for redox, light and oxygen status, although they may also participate in ligand binding (Henry and Crosson, 2011). Rup4959 is an orphan response regulator and the presence of the receiver REC and PAS domains suggest that Rup4959 likely requires phosphorylation and may respond to intracellular and/or environmental signals for its functionality.

A significant increase in c-di-GMP levels can be observed in *P. putida* when *rup4959* is present in a multicopy plasmid, which is accompanied by a pleiotropic phenotype that includes increased biofilm formation on surfaces, pellicle formation on the liquid-air interface and bacterial flocculation in liquid cultures, as well as the development of crinkly colonies on solid medium. By analyzing available mutants that we suspected might be hampered in this pleiotropic phenotype, we unveiled that its appearance requires lipopolysaccharides (LPSs) and the *P. putida*-specific EPS Pea. Additionally this phenotype is dependent on the stationary phase sigma factor RpoS (Matilla et al., 2011), and as such, the phenotype becomes apparent in cultures at late growth stages and in cultures streaked on solid medium (**Supplementary Figure S1**). Interestingly, the appearance of phenotypes like flocculation and increased biofilm formation can be accelerated by the addition of spent supernatants of KT2440 cultures (Matilla et al., 2011), suggesting that some extracellular signal or cell lysate products could be contributing to c-di-GMP accumulation. There is also evidence that not all the previously mentioned phenotypes are fully associated. Thus a mutant in the gene coding the main *P. putida* adhesin, LapA, loses the flocculation ability and is unable to develop biofilms in the presence of multicopy *rup4959*, although still retains the crinkly colony morphology on agar plates (Matilla et al., 2011; Martínez-Gil et al., 2014).

In this work we aimed to decipher the genetic network associated with high c-di-GMP levels and the pleiotropic phenotype in *P. putida*. We generated two independent mutant libraries and high-throughput screens were designed to identify genes either involved in the signaling pathway leading to, or required for, the pleiotropic phenotype associated with overexpression of the DGC Rup4959.

## Materials and Methods

### Bacterial Strains, Culture Media and Growth Conditions

*Pseudomonas putida* strains were grown at room temperature, 28 or 30°C as indicated, in either Luria-Bertani (LB) medium (Bertani, 1951) or M9 minimal medium (Sambrook et al., 1989) supplemented with 1 mM MgSO_4_, 6 μg/L ammonium ferric citrate and trace metals as described previously (Yousef-Coronado et al., 2008). Glucose (27 mM) or sodium citrate (15 mM) were added as alternative carbon sources to defined M9-minimal medium. *Escherichia coli* strains were grown at 37°C in LB. Bacterial strains and plasmids used in this work are listed in **Table 1**. When appropriate, antibiotics were added to the medium at the following final concentrations (μg/ml): ampicillin, 100 (*E. coli*); chloramphenicol, 30; gentamicin, 10 (*E. coli*), and 50 or 100 as indicated (*P. putida*); kanamycin 25; nitrofurantoine (NF), 100; piperacillin 30, routinely, or 50 (for counter selection of *P. putida*); rifampin, 10; streptomycin, 50 (*E. coli*), and 100 (*P. putida*); tetracycline, 7.5. To induce gene expression in *P. putida* from expression vectors IPTG 0.1 mM was used.

**Table 1:** Bacteria and plasmids used.

Strains and plasmids	Relevant characteristics^a^	Reference or source
*E. coli* DH5α	*lacZΔ M15*, *recA1, endA1*	Woodcock et al., 1989


*E. coli* HB101 (pRK600)	Cm^R^, transfer functions helper strain	V. DeLorenzo


*E. coli* S17-1 λ*pir*	Sm^R^, *λpir* RP4 transfer functions	Simon et al., 1983


*P. putida* KT2440	Derivative of *P. putida* mt-2, cured of pWWO	Regenhardt et al., 2002


*P. putida* KT2440R	Rif^R^, derivative of KT2440 mutated in the RNAP β subunit	Espinosa-Urgel and Ramos, 2004


Plasmids		


pBBR1-MCS2	Km^R^, broad host range cloning vector, mobilizable	Kovach et al., 1995


pBBR1-MCS5	Gm^R^, broad host range cloning vector, mobilizable	Kovach et al., 1995


pCAlgU1-75	Tc^R^, mob*RK2*, derivative cosmid of pLAFR3 with a KT2440 DNA insertion of 27.6 kb including *algU* (coordinates 1.626.675-1.654.289)	This work


pCdrA::*gfp*^C^	Ap^r^, Gm^R^, FleQ dependent c-di-GMP biosensor	Rybtke et al., 2012


pCFleQ1-18	Tc^R^, mob*RK2*, derivative cosmid of pLAFR3 with a KT2440 DNA insertion of 21.2 kb including *fleQ* (coordinates 4.952.619 -4.973.853)	This work


pMAMV1	Gm^R^, derivative plasmid of pBBR1-MCS5 containing *rup4959* of *P. putida* KT2440 and it is expressed from its own promoter; it confers high c-di-GMP levels in the wt strain	Matilla et al., 2011
pME1088	Ap^R^ (Pip^R^), derivative plasmid of pMMB67HE for the ectopic expression of *P. putida argG*	This work
pME0184	Ap^R^ (Pip^R^), derivative plasmid of pMMB67HE for the ectopic expression of *P. putida argH*	This work
pMIR178	Km^R^, derivative plasmid of pBBR1-MCS2 containing *rup4959* of *P. putida* KT2440 and it is expressed from its own promoter; it confers high c-di-GMP levels in the wt strain	M. I. Ramos-González
pMIR190	Gm^R^, derivative of pMAMV1 encoding Rup4959 inactive (D65→A65)	This work
pMIR203	Ap^R^ (Pip^R^), *gacA* expression plasmid derivative of pMMB67EH	This work
pMIR205	Sm^R^, derivative plasmid of pSEVA443 containing *cfcA* of *P. putida* KT2440 in a 6.1 kb-BsrGI fragment, cloned from a recombinant cosmid, at its Acc65I site	This work
pMIR216	Sm^R^, *algU* expression plasmid derivative of pSEVA443	This work
pMIR231	Sm^R^, derivative plasmid of pMIR205 after removing downstream genes to *cfcA* with ScaI/NdeI. It harbors *cfcA* with its own promoter	This work
pMIR232	Sm^R^, derivative plasmid of pMIR205 encoding inactive CfcA (H496→A496)	This work
pMMB67EH	Ap^R^ (Pip^R^), RSF1010 origin, *lacI^q^*	Fürste et al., 1986
pMMB67HE	Ap^R^ (Pip^R^), RSF1010 origin, *lacI^q^*	Fürste et al., 1986
pSEVA443	Sm^R^, cloning vector with ColE1 and pRO1600 origin combination, *lacZα* complementation	V. DeLorenzo
pUTkm1	Ap^R^, Km^R^, *oriR6K mobRK2*, mini-Tn5[Km1] transposon delivery vector	DeLorenzo et al., 1990
pUT-Tc	Ap^R^, Tc^R^, *oriR6K mobRK2*, mini-Tn*5*[ΩTc] transposon delivery vector	DeLorenzo et al., 1990


*Ap, ampicillin; Cm, chloramphenicol; Gm, gentamicin; Km, kanamycin; Pip, piperaciline; Rif, rifampicin; Sm, streptomycin; Tc, tetracycline.*

### Random Transposon Mutagenesis

Transposon mutagenesis was performed by mating with mini-Tn*5*[Km1], or mini-Tn*5*[ΩTc], two mini-Tn*5* derivatives carrying respectively a kanamycin or tetracycline resistance marker (DeLorenzo et al., 1990). The recipient (*P. putida* KT2440) and donor (*E. coli* S17-1 λ*pir* harboring pUTkm1 or alternatively pUT-Tc, the suicide vectors carrying mini-Tn*5*[Km1] or mini-Tn*5*[ΩTc], respectively), were grown overnight in LB with the appropriate antibiotics. A volume of 0.75 ml of each strain was mixed and cells were collected by centrifugation, then rinsed and resuspended in 50 μl of fresh LB, and finally spotted on a filter of 0.45 μm of pore diameter. Up to 10 independent conjugations were performed in parallel to maximize heterogeneity and minimize siblings in the mutant library. After overnight incubation at 30°C, cells were scraped off the mating filter and resuspended in 10 ml of M9 buffer, and serial dilutions were plated on selective LB medium supplied with Km or Tc plus NF to counterselect the *E. coli* donor strain. Two independent mutant libraries were generated, one based on mini-Tn*5*[Km1] and the other based on mini-Tn*5*[ΩTc].

All selected mutants were verified by Southern-blot to have single insertions of the mini-Tn*5* derivatives using specific probes against Km or Tc resistance-encoding genes. (Primers used in this work are listed in **Supplementary Table S1**).

### *In Vitro* Nucleic Acid Techniques

Total DNA extraction was performed using the Wizard^®^ genomic DNA purification kit (Promega Cat. No. A1120). Plasmid DNA was isolated using the Quantum Prep Plasmid Miniprep Kit (Biorad Cat. No. 732-6100). For DNA digestion, the manufacturer’s instructions were followed (Roche and New England Biolabs). Separated DNA fragments were recovered from agarose using the high pure PCR cleanup micro kit (Roche Cat No. 04983955001). Alkaline phosphatase, ligation reactions, Southern blots and colony hybridization were performed by standard protocols (Sambrook et al., 1989). DNA digoxigenin-dUTP probes were obtained via PCR following the instructions of the manufacturer (Roche). The Expand high fidelity PCR system (Roche Cat. No. 11732641001) was used for the amplification of PCR fragments for cloning. Sequences of these PCR fragments were verified in order to discard amplicons containing mutations. Competent cells were prepared using calcium chloride and transformations were performed by standard protocols (Sambrook et al., 1989). Highly electrocompetent cells were prepared as previously reported (Choi et al., 2006) and transformed using an EC100 electroporator according to the manufacturer’s instructions (EC apparatus corporation).

### Plasmid Conjugation and Plasmid Curing Experiments

Transfer of pMAMV1 or pMIR178 from *E. coli* DH5α to the transposon insertional mutants of *P. putida* was performed in mass by triparental matings using *E. coli* (pRK600) as a helper. Filter matings were used as described in the previous section.

Phenotypes of the exconjugants (crinkly morphology, flocculation and biofilm formation) were analyzed as specified below.

Mutants of interest were cured of their plasmid by removing the selective antibiotic, Gm or Km, from the culture media followed by screening for antibiotic sensitive colonies. After plasmid extraction antibiotic sensitive colonies were confirmed to be free of plasmids.

Once strains were cured of plasmid, pMAMV1 or pMIR178 were newly transferred to the transposon insertional mutant strains by triparental mating as described above. These plasmids were transferred by conjugation because electrotransformation of *P. putida* cells caused rearrangement within these plasmids, which was not observed when they were transferred by conjugation. At least 50 exconjugant colonies were tested per strain to confirm their phenotype.

### Identification of the Transposon Insertion Site

For Mini-Tn5[Km1] insertional mutants, arbitrary PCR was used following the previously described method (O’Toole and Kolter, 1998). Arbitrary primers ARB1, ARB6 or ARB1-1-6 were alternatively used with the specific primer TNEXT in the first PCR round. Primer ARB2 was used with primer TNINT in the second PCR round. Further sequencing of the PCR amplicons was performed with oligonucleotides TNINT and TNOEND. For the mutants containing the mini-Tn5[ΩTc], genome sequencing from both ends of the mini transposon was carried out using primers Tn5Iend or Tnint1end and TnTc0.

### Construction of Recombinant Expression Plasmids

Recombinant cosmids pCAlgU1-75, pCCfcA18-7 and pCFleQ1-18 were isolated from a genome library of *P. putida* KT2440 (Ramos-González, 1993) using specific probes against *algU*, *cfcA* and *fleQ* respectively. Primers used to amplify probes and to amplify genes for their expression are listed in Table S1. PCR amplicons were firstly cloned in pGEM-T and then sequenced to verify that they were free of mutations.

Plasmid pMIR203 was generated by cloning a 650 bp fragment containing the *gacA* gene from KT2440 amplified with oligos GacAFEco and GacARSal, using the EcoRI/SalI sites of the expression vector pMMB67EH. Plasmid pMIR205 was generated by cloning a 6.1 kb-BsrGI fragment containing *cfcA* from cosmid pCCfcA18-7 at the Acc65I site of the expression vector pSEVA443. Plasmid pMIR216 was generated by cloning a 580 bp fragment containing the *algU* gene from KT2440 amplified with oligos ALGU2FW and ALGU2RV, at the SphI and EcoRI sites of the expression vector pSEVA443. Plasmids pME1088 and pME184 were constructed by cloning 1.2 and 1.4 kb HindIII/SacI fragments containing the coding regions of *argG* and *argH* into expression vector pMMB67HE, after amplification with primers ARGG-F/ARGG-R and ARGH-F/ARGH-R, respectively. In all cases, the constructs were sequenced (both strands), to check the absence of mutations.

### Site Directed Mutagenesis of D_65_ in the Diguanilate Cyclase Rup4959

The aspartic acid residue present in Rup4959 at position 65 (D65) was replaced with alanine (A65). For the mutation of the codon encoding D65 in pMAMV1, one amplicon was obtained by Expand High Fidelity PCR system (Roche) with the pair of primers pMAMV1-SalI (AGTCGACCTGGTGTTGATG*G**C**T* GTA) and pMAMV1-MfeIRev (CACAACGTCGAGCAATTGGG). Restriction sites are underlined. The codon for A65 is underlined and italicized and the base cytosine mutated to attain this residue is in bold. A further amplicon of 436 bp was obtained which was cloned in pGEM-T generating pMIR189. The wild type SalI/MfeI fragment in pMAMV1 was replaced with that of pMIR189 to generate pMIR190. The replacement of the wild type sequence GAT with GCT in pMIR189, as well as the absence of further mutations, were confirmed by sequencing.

### Site Directed Mutagenesis of His496 in PP_3761

The histidine residue present in PP_3761 at position 496 (H496) was replaced with alanine (A496). An intermediate construction, plasmid pMIR231, was first generated by removing from pMIR205 the genes downstream from the *orf* encoding PP_3761 in a 2 Kb ScaI/NdeI fragment and further re-ligation of the 8 Kb ScaI/NdeI fragment corresponding to pSEVA443 plus the *orf* encoding PP_3761 under the control of its own promoter. For the mutation of the codon encoding H496, overlapping PCR was performed with Q5^®^ High-Fidelity DNA Polymerase (New England Biolabs M0491). Upstream and downstream amplicons were obtained with the pairs of primers CfcA-OutF 5′GAGAGTGCCCGTTATCGCCAG3′/CfcA-AlaR 5′GC GCAGCTC*A**GC***GGACATGTTG3′ and CfcA-AlaF 5′GCCAAC ATGTCC***GC**T* GAGCTGC3′/CfcA-OutR 5′GCTGAACACGCT GCCTTGC3′, respectively. These oligonucleotides were designed to replace the codon CAT (His496) with the codon GCT (Ala496). Oligo CfcA-SbfIF 5′GCCTGCAGGAAGTGCTGGC3′, which anneals internal to the upstream amplicon, and oligo CfcA-SalIR 5′CTGCTGTCGACATTGATCTGGC3′, which anneals internal to the downstream amplicon, were further used for the amplification of 910 bp containing the mutated codon. This PCR amplicon was cloned at the EcoRV site of circular pCR2.1-TOPO generating pMIR229. Restriction sites are underlined. The codon for A496 is underlined and italicized and the bases GC mutated to attain this residue are in bold. The wild type SbflI/SalI fragment in pMIR231 was replaced with that of pMIR229 and pMIR232 was generated. The replacement of the wild type sequence CAT with GCT in pMIR232, as well as the absence of further mutations, were confirmed by sequencing.

### Crinkly Morphology and Flocculation Analysis

Pictures of the bacterial streaks were taken after 24 or 48 h incubation as indicated. Pictures of flocculation were taken after incubation for 16 h under orbital shaking (200 rpm) followed by static incubation on the bench for 30 min.

### Biofilm Assays

Biofilm formation assays were performed in borosilicate tubes that were incubated in an orbital shaker (40 rpm) with bacterial cultures grown in LB medium or glucose-M9 medium supplemented with Casaminoacids 0.5% (w/v) as described before (Matilla et al., 2011). The staining solution (crystal violet solution, HT90132 Sigma) was discarded and the stained tubes were washed three times with water, prior to analysis as previously reported (O’Toole and Kolter, 1998).

### Extraction and Quantification of c-di-GMP

For the extraction of nucleotides from the bacteria, cells were grown in LB until early stationary phase of growth to allow *rup4959* expression. Cells corresponding to 7 ml of culture were then lysed by heat and the nucleotides extracted twice, as previously described, with 65% ethanol (Matilla et al., 2011). Nucleotide extracts were filtered through a polyvinylidene fluoride (PVDF) membrane (0.2 μm pore size; PALL Life Sciences Cat. No. 4555) and the samples were analyzed using reverse phase-coupled HPLC-MS/MS. High performance liquid chromatography (HPLC) separation was carried out on an Agilent 1100 Series instrument using a 12 × 3 mm reverse phase column (Waters Spherisorb ODS2, 5 μm particle size; Cat. No. PSS838529) with UV detection at 248 nm. Samples were loaded onto the column which had been equilibrated with an aqueous solution of formic acid (0.1%; v/v). Compounds were eluted with a linear gradient of acetonitrile 0% to 30% (0.5 ml/min) over 16 min followed by a linear increase to 90% of acetonitrile in 3 min. To eliminate carryover between injections, 0.1% formic acid was then increased linearly to 100% in 2 min, followed by a decrease to 10% in 5 min and finally restored to 100% in 2 min. C-di-GMP was detected by MS/MS with an Esquire 6000 mass spectrometer (Bruker Daltonics) using positive electrospray ionization. The fragmentation of c-di-GMP resulted in three main ions with mass to charge rate (m/z) of 540, 248 and 152, as previously described (Simm et al., 2004). C-di-GMP quantification in the cellular extracts was estimated by calculating the peak areas of the ion with m/z 248. The concentrations were further extrapolated from a standard curve obtained with synthetic c-di-GMP (BioLog Cat. No. C-057, Bremen, Germany) in the range between 31.25 and 2000 nM. Two independent biological replicates, with two technical replicates each, were analyzed. Our limit of detection was around 30 nM of c-di-GMP. Final concentrations were expressed as pmol of c-di-GMP per mg of bacterial protein. Protein content was determined as previously described (Spangler et al., 2010) using the Bradford assay (Bio-Rad laboratories, Munich, Germany).

### Bioreporter-Based c-di-GMP Measurement Assays in Microtiter Plates

Indirect detection of c-di-GMP contents was carried out by microtiter plate-based assays of the strains with the reporter pCdrA::*gfp*^C^ using Greiner 96 black-welled plates. Overnight cultures grown in LB were diluted in 1/10 LB to an OD_600 nm_ of 0.05 in the presence of 20 μg/ml gentamicin (final volume of 200 μL). Growth and fluorescence (excited at 485 nm and read at 535 nm) were monitored in an Infinite 200 Tecan fluorometer. The experiments were conducted in triplicate over 24 h, taking measures every 30 min.

## Results

### Isolation of cfcK and cfcT Strains Defective in the Crinkly Colony Phenotype Associated to High c-di-GMP Levels

**FIGURE 1:**
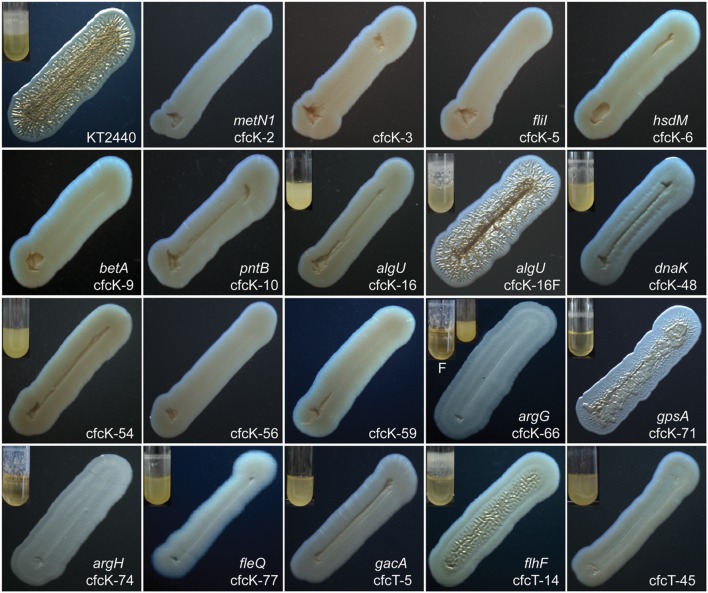
**Effect of over expressing diguanylate cyclase Rup4949 upon streak morphology of the wild type and cfc derivatives of *Pseudomonas putida*.** All strains harbor pMAMV1. LB-agar plates supplemented with Gm 50 μg/mL were incubated at 30°C for 48 h and pictures were taken using Leika stereomicroscope M165FC. Small inserts show biofilms of selected strains (those further characterized and those partially keeping crinkly morphology) formed after 24 h under rotation (40 rpm); the rest do not form biofilm after 24 h and thus are not shown for simplicity. Two variants of cfcK-16 harboring pMAMV1 are designated cfcK-16 and cfcK-16F (see **Table 2**). Two inserts corresponding to two non-crinkly variants of cfcK-66 harboring pMAMV1 are shown together with a unique microscope image, which was similar for both variants; the flocculating and non-flocculating variants are designated cfcK-66 (pMAMV1)F and cfcK-66 (pMAMV1), respectively.

A genetic screen was designed to identify, by visual inspection, mutants that did not form crinkly streaks after introducing the medium-copy number plasmid pMAMV1 harboring the gene *rup4959* of *Pseudomonas putida* (**Table 1**). Mini-Tn*5*[Km1] was used for random mutagenesis of KT2440. The resulting Km^R^ clones were pooled and en-masse mating experiments were used to transfer pMAMV1 to the mutants. About fifteen thousand ex-conjugant clones were then streaked onto LB-agar plates and their morphology visually analyzed. Approximately eighty clones were initially isolated as having lost the crinkly colony morphology despite carrying extra copies of *rup4959*. These mutants were termed cfcK (for crinkle-free colony/Km^R^). Selected mutants were cured of plasmid pMAMV1 and the same plasmid was newly transferred into them by conjugation in order to confirm their cfc phenotype and to analyze the incidence of this phenotype in a greater number of isolates of the mutant strain populations. In all cases the presence of the intact pMAMV1 plasmid was confirmed, ensuring that the loss of the phenotype was not due to plasmid deletions or rearrangements. Results are summarized in **Figure 1**. In addition to morphology, we also analyzed culture flocculation (clumps of bacterial cells coming out of suspension) and biofilm formation in all the cfcK mutants (**Table 2**). The gene disrupted in each case was identified by arbitrary PCR from the genome as described in Section “Materials and Methods”. Several of the cfcK mutants turned out to be siblings. In particular, it is noticeable that nine clones harbored the transposon insertion in the last codon of the *algU* gene, just before the stop codon, although they were in two different positions (termed cfcK-16 in **Table 2**). When re-transformed by conjugation with pMAMV1 as reported above, only a subpopulation of cfcK-16 carrying pMAMV1 showed the cfc phenotype. The other subpopulation was given the name cfcK-16F (pMAMV1) (**Figure 1**; **Table 2**). By contrast, an independent transposon insertional inactivation located in the core of the *algU* gene did not cause any mutant phenotype (not shown). This result was consistent in all the clones and indicates that the loss of functionality of *algU* was probably not the reason for the crinkle-free phenotype of the variant cfcK-16 (pMAMV1). Two other transcriptional regulators, PP_4630 and FleQ (cfcK-77), were also identified in this screen. The later has been previously reported to function in association with c-di-GMP in *P. aeruginosa* (Hickman and Harwood, 2008). The binding of this second messenger to the Walker A motif in the AAA+ conserved domain of FleQ has also been demonstrated in this bacterium (Baraquet and Harwood, 2013). In addition, three other molecular determinants with AAA+ ATPase domains were identified in this screen: the ATP synthase FliI, the ATP-binding protein MetN1 (a component of the methionine import transporter), and a putative GTP-binding protein encoded by the locus PP_0015. In two other mutants the locus inactivated was PP_3761 (cfck-54), which encodes a hybrid sensor histidine kinase (HK). Three additional mutants were defective in *argG* (cfcK-66) and *argH* (cfck-74), which encode enzymes acting sequentially in the arginine biosynthetic pathway (**Supplementary Figure S2**). Interestingly these mutants still retained the flocculation phenotype and were only partially hampered in biofilm formation capacity when carrying pMAMV1. Several other genes involved in metabolism were also identified in this screen, i.e., *gpsA* (encoding a glycerol-3-P dehydrogenase) and *betA* (encoding a choline dehydrogenase). When examined under the stereomicroscope only mutants in the glycerol-3-P dehydrogenase and the chaperone protein DnaK kept partial crinkle phenotype (**Figure 1**).

**Table 2:** Characteristics of cfcK mutants.

Strain^a^	Crinkly morphology	Flocculation	Increased biofilm	Locus (gene)	Function and characteristics
KT2440	++	++	++		
cfcK-2	–	–	–	PP_0114 (*metN1*)	Methionine import ATP-binding component of ABC transporter (AAA+ ATPase domain)
cfcK-3	–	–	–	PP_5322	Putative metal ion transporter
cfcK-5	–	–	–	PP_4366 (*fliI*)	Flagellum-specific ATP synthase (AAA+ ATPase domain)
cfcK-6	–	–	–	PP_4741 (*hsdM*)	Type I restriction-modification system, M subunit
cfcK-9	–	–	–	PP_5064 (*betA*)	Choline dehydrogenase
cfcK-10	–	–	–	PP_0155 (*pntB*)	NAD(P) transhydrogenase subunit beta
cfcK-16 (9)	-/++^b^	-/++^b^	-/++^b^	PP_1427 (*algU*)	Alternative σ-factor
cfcK-48	+^c^	–	+	PP_4727 (*dnaK*)	Chaperone
cfcK-54 (2)	–	–	–	PP_3761	Multisensor hybrid histidine kinase
cfcK-56	–	–	–	PP_4630	Transcriptinal regulator MerR family
cfcK-59	–	–	–	PP_0015	Putative GTP-binding protein (AAA+ ATPase domain)
cfcK-66	–^d^	++/-^e^	+/-^e^	PP_1088 (*argG*)	Argininosuccinate synthase
cfcK-71	+	++	+	PP_4169 (*gpsA*)	NAD(P)H-dependent glycerol-3-phosphate dehydrogenase
cfcK-74 (2)	-^d^	++	+	PP_0184 (*argH*)	Argininosuccinate lyase
cfcK-77	–	–	+	PP_4373 (*fleQ*)	Transcriptional regulator (AAA+ ATPase domain)


*The number in parentheses indicates the number of mutants in the case that more than one were isolated in the same locus. ^a^The phenotype indicated corresponds to the strains bearing pMAMV1, wild type included, which harbors multiple copies of *rup4959* compared to the wild type parent strain (negative for the three phenotypes). Floccules and biofilm were visualized in LB after 24 h under orbital shaking at 200 rpm; crinkly phenotype after 48 h. ^b^Although initially isolated as non-crinkly, when cured and newly transformed with pMAMV1, clones showing two different phenotypes were observed [cfcK-16 (pMAMV1) and cfcK-16 (pMAMV1)F, see **Figure 1**]. ^c^Crinkly morphology only noticeable under stereomicroscope, see **Figure 1**. ^d^Non-crinkly modified morphology, noticeable as an apparent “dried phenotype”, see **Figure 1**. ^e^Although initially the mutant was isolated as non-crinkly and formed flocculates, when cured from the plasmid and newly transformed with pMAMV1, two variants of non-crinkly clones were observed [cfcK-66 (pMAMV1)F and cfcK-66 (pMAMV1), see **Figure 1**].*

To increase mutants heterogeneity a smaller gene library based on mini-Tn*5*[ΩTc] was also generated and analyzed by en-masse transfer of plasmid pMIR178. This plasmid confers kanamycin resistance but otherwise carries the same insert as pMAMV1 encoding Rup4959. Mutants in *gacA*, *flhF*, *argH* and in one of the seven copies for RNA23S present in *P. putida* KT2440 were isolated as crinkle-free colonies when carrying pMIR178 (**Figure 1**); these strains were designated cfcT (for crinkle-free colony/TcR, **Table 3**). It was not possible to discern which of three possible RNA23S was affected in cfcT-45, and this mutant was not studied further. Like FleQ, the flagellar biosynthetic protein FlhF also contains a Walker A motif. The two component system GacS/GacA is considered a master regulator of *Pseudomonas* secondary metabolism (Heeb and Haas, 2001; Wei et al., 2013) and regulates the transcription of RpoS in KT2440 (Martínez-Gil et al., 2014), which is essential for *rup4959* expression (Matilla et al., 2011). It should be noted that an *argH* minus strain was also identified as a cfcK strain in the screening performed with the gene library based on mini-Tn5[Km1] (cfcK-74 in **Table 2**).

**Table 3:** Characteristics of cfcT mutants.

Strain^a^	Crinkly morphology	Floccules	Increased biofilm	Locus (gene)	Function
KT2440	++	++	++		
cfcT-5	–	–	–	PP_4099 (*gacA*)	Transcriptional regulator of the two component system GacS/GacA
cfcT-12	–^b^	++	++	PP_0184 (*argH*)	Argininosuccinate lyase
cfcT-14	+	++	++	PP_4343 (*flhF*)	Flagellar biosynthesis regulator (AAA+ ATPase domain)
cfcT-45	–	–	–	Uncertain^c^	23S rRNA


*^a^The phenotype indicated corresponds to the strain carrying pMIR178, which harbors multiple copies of *rup4959*. ^b^Modified colony morphology (“dried phenotype”, see **Figure 1**). ^c^The site of insertion of the transposon did not allow to discern between three of the seven copies of 23S rDNA to define which one was interrupted.*

### Characterization and Complementation of cfc Mutants

None of the cfc mutants were hampered in growth, either in rich LB or defined media, except for cfcT-5 and cfcK-16, which exhibited a reduced lag phase in all media tested, whereas this stage was slightly longer in cfcK-54. Most of the crinkle-free colony mutants did not show any flocculation, while a few kept somewhat increased biofilm formation as compared to the wild type parent strain (free of plasmid pMAMV1 or pMIR178) which was negative for the three phenotypes (**Table 2**). To ensure the consistency of these phenotypes, cfcK mutants were cured of plasmid pMAMV1, and then the plasmid was reintroduced and the phenotypes reassessed in a minimum of 50 exconjugants.

**FIGURE 2:**
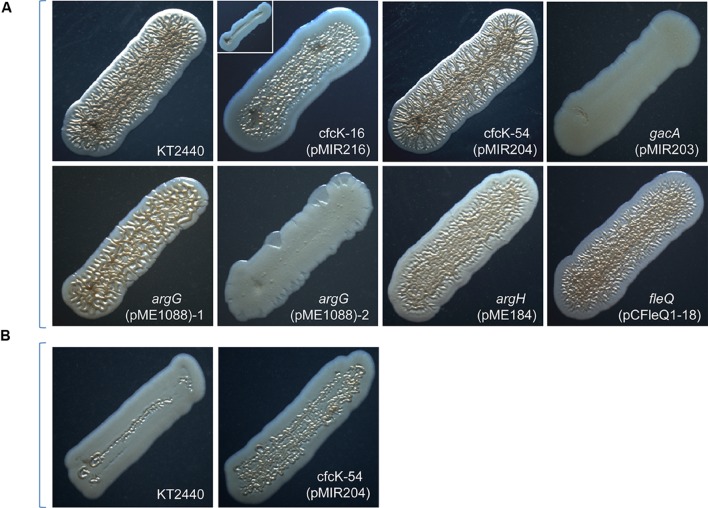
**Morphology of cfcK and cfcT mutants after genetic complementation of their knocked out loci with the corresponding wild type alleles.** All strains harbor pMAMV1 except *gacA* which harbors pMIR178. **(A)** Pictures were taken as indicated in the legend for **Figure 1** after 48 h of incubation. Two varieties observed for cfcK-16 harboring the plasmid pMIR216 (with *algU* wt allele) and pMAMV1 are shown in the same panel. Nomenclature cfcK-16 is kept since other inactivation of *algU* did not cause the phenotype of cfcK-16 mutant (see the text for details). About 20% percent of cfcK-66/*argG* colonies that harbored pMIR1088 (with *argG* wt allele) and pMAMV1 exhibited a similar phenotype to that shown in the panel for *argG* (pMIR1088)-1 and the rest similar to that of the panel *argG* (pMIR1088)-2. **(B)** Pictures were taken after 24 h.

To check whether the incorporation of the intact gene restored the phenotypes associated with high c-di-GMP in crinkle-free colony mutants, two approaches were used: either, using a recombinant cosmid isolated from a gene library of KT2440 carrying the corresponding wild type allele, or by PCR amplification of the knocked-out gene and subsequent cloning in an expression vector. A selection of mutants were chosen for the complementation analyses: cfcT-5 (*gacA*), cfcK-16 (*algU*), and cfcK-77 (*fleQ*), which allowed to investigate the significance of the transcriptional regulators on *rup4959* expression; cfcK-66 (*argG*) and cfcK-74 (*argH*), both hampered in the arginine biosynthesis pathway; and cfcK-54 (PP_3761), the unique mutant inactivated in a HK, which together with a response regulator are the elements of a two-component phosphorelay system. For the complementation assays, each plasmid/cosmid was introduced into the corresponding cfc strain by conjugation. Once the presence of these plasmids, and that of pMIR178 or pMAMV1, was confirmed then colony morphology, flocculation and increased biofilm formation were re-analyzed. The results from these studies are summarized in **Table 4** and shown in **Figure 2**. Complementation was observed for cfcK-54, cfck-74, and cfcK-77, although in the later two the crinkle phenotype did not reach the border of the streak. For mutant cfcT-5 crinkly morphology was not restored although biofilm formation was partially and flocculation completely restored. After receiving their wild type alleles mutants cfcK-16 and cfcK-66 both showed subpopulations with nearly full complementation and others presented the non-crinkly morphology (**Figure 2A**). Interestingly complementation of cfcK-54 could be observed after 24 h incubation whereas crinkly morphology was better observed in the wild type only after incubation for 48 h (**Figure 2B**).

**Table 4:** Complementation patterns of selected cfc mutants.

Strain^a^	Crinkly morphology	Flocculation	Increased biofilm
KT2440 (pMAMV1)	++	++	++
KT2440 (pMIR178)	++	++	++
cfcT-5 (pMIR203) (pMIR178)	–	++	+
cfcK-16 (pCAlgU1-75) (pMAMV1)	-/+^b^	++	++
cfcK-16 (pMIR216) (pMAMV1)	-/+^b^	++	++
cfcK-54 (pMAMV1) (pMIR205)	++^c^	++	++
cfcK-66 (pME1088) (pMAMV1)^d^	++/-	++	++
cfcK-74 (pME184) (pMAMV1)	+	++	++
cfcK-77 (pCFleQ1-18) (pMAMV1)	+	++	++


*^a^The loci inactivated in the strain mutants are indicated in **Tables 2** and **3**.*

*^b^Incorporation of a wild type allele in the non-crinkly mutant complemented flocculation and biofilm formation in 100% of clones; crinkly morphology, however, was partially restored only in a limited number of clones (see **Figure 2**). ^c^Crinkly phenotype appears after 24 h, earlier than in the wild type, which is evident after 48 h (see **Figure 2**). ^d^About 20% showed a phenotype similar to the wild type and the rest shows a non-crinkly phenotype although modified (see **Figure 2**).*

### c-di-GMP Levels in cfc Mutants

**FIGURE 3:**
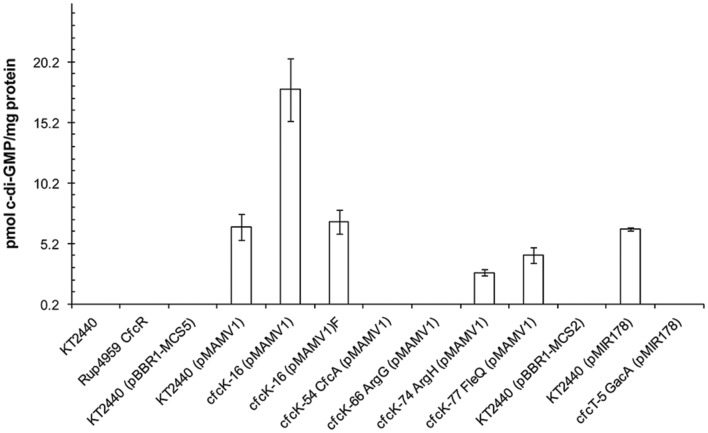
**Relative c-di-GMP content in *P. putida* strains.** Bacteria were cultivated in LB until the stationary phase of growth (OD_660 nm_ = 2.8). Extraction was carried out as described in Section “Materials and Methods”. Samples were analyzed using reverse phase-coupled HPLC-MS/MS. Data derived from the average and standard deviation from two independent biological replicates, with two technical replicates each, are shown. Nomenclature cfcK-16 is kept since other inactivation of *algU* did not cause the phenotype of cfcK-16 mutant (see the text for details). CfcR and CfcA are the proposed name for Rup4959 and PP_3761 (see Discussion). Differences between KT2440 (pMAMV1) and cfcK-16 (pMAMV1), *argH* (pMAMV1) and *fleQ* (pMAMV1) are statistically significant (Student’s *t*-test, *P* < 0.01). Values below detection limit (∼ 2 pmol mg^-1^ of bacterial protein) are not plotted.

To determine if the loss of the crinkle phenotype in the cfc mutants harboring pMAMV1 or pMIR178 was due to their reduced c-di-GMP levels, as a consequence of a diminished expression and/or activity of the DGC Rup4959, or to an impaired transduction of the second messenger signal to downstream processes required for the phenotype (such as synthesis of EPS or adhesins), total c-di-GMP intracellular levels were measured (**Figure 3**). As previously reported, specific levels of the second messenger were under the detection limit in *P. putida* KT2440 and those strains harboring expression vectors (Matilla et al., 2011) whereas pMAMV1 or pMIR178 conferred to this strain augmented and therefore measurable levels of c-di-GMP. The results show that the plasmids did not increase c-di-GMP of *gacA*, PP_3761 and *argG* knock-out mutants up to detectable levels. Given that *rpoS* expression is entirely dependent on GacA in *P. putida* (Martínez-Gil et al., 2014), and that *rup4959* is in turn completely dependent on RpoS (Matilla et al., 2011) we can conclude that the undetectable c-di-GMP values of *gacA* (pMIR178) were due to a loss of *rup4959* expression (**Figure 3**). In addition, it is tempting to speculate that the HK encoded by PP_3761 might be required to phosphorylate and activate the REC domain of Rup4959. Thus a strain with a mutation in this kinase (PP_3761) carrying pMAMV1 would present an increased level of an unphosphorylated and thus inactive form of Rup4959, which explains why the plasmid did not augment the levels of c-di-GMP in this particular genetic background. We have confirmed that mutants *argG* and *argH* are both auxotrophic for arginine (**Supplementary Figure S3**). However, the mutant *argH*, unlike *argG*, presented detectable c-di-GMP levels although they were significantly lower than those of the wild type when both strains carried pMAMV1 (**Figure 3**). The altered second messenger content in these mutants was not due to a modification in the *rup4959* expression pattern (**Supplementary Figure S4**). Besides c-di-GMP values of *fleQ* when harboring pMAMV1 presented 40% reduction compared to the wild type. This decrease can be explained by the reduced expression of *rup4959* found in a *fleQ* background (40% level of expression in the mutant compared to the wild type at the beginning of the stationary phase, a difference which was maintained in later stages; **Supplementary Figure S5A**). Interestingly, a reduction of *rup4959* expression was also detected in the cfcK-16 genetic background (**Supplementary Figure S5B**) indicating that FleQ and AlgU/MucA are in the regulation cascade of *rup4959.* These two elements have been postulated to be part of the GacS/GacA regulatory cascade in *P. fluorescens* F113 (Martínez-Granero et al., 2012). Finally, the values of the second messenger were detectable in both variants of cfcK-16 harboring pMAMV1, although they greatly differed between each variant (**Figure 3**); interestingly, the non-flocculating variant strain showed the highest level of c-di-GMP which cannot be explained by the negative effect of cfcK-16 upon *rup4959* expression. Furthermore this latter strain showed slight reduced motility (data not shown), a state that usually corresponds with increased c-di-GMP (Römling et al., 2013).

**FIGURE 4:**
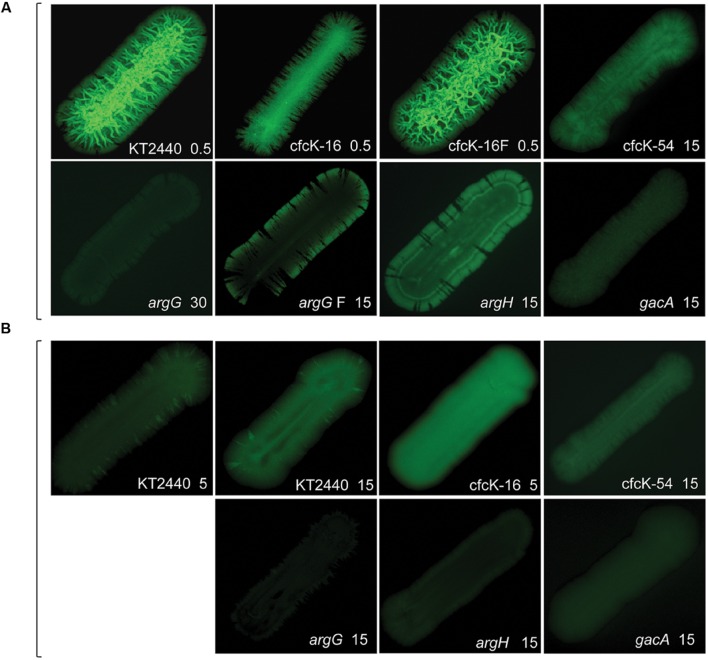
**Fluorescence emitted by *P. putida* strains harboring the pCdrA::*gfp*^C^ plasmid.** In each case the number next to the name of the strain indicates exposure time elapsed in seconds. Bacteria were cultivated as in the legend for **Figure 1** and pictures of the dark fields were taken with a Leika M165FC stereomicroscope using Excitation/Emission filter 494/518 nm. **(A)** Diguanylate cyclase overexpression was rendered by pMAMV1 in KT2440 and cfcK strains, and by pMIR178 in cfcT-5/*gacA*. Plasmids pMAMV1 and pMIR178 are derived from cloning vectors pBBR1-MCS5 and pBBR1-MCS2, respectively. Two variants of cfcK-16 (pMAMV1) and cfcK-66/*argG* (pMAMV1) are shown. A description of these variants is made in the text and in the legends to **Figure 2** and **Table 2**. **(B)** KT2440 and cfcK strains carry vector pBBR1-MCS5 and cfcT-5/*gacA* carries pBBR1-MCS2.

Given the difficulty in detecting c-di-GMP in several strains using our standard analytic method (**Figure 3**) we used an alternative technique to indirectly detect free second messenger with the biosensor based on the promoter of *cdrA* fused to *gfp*^C^ (Rybtke et al., 2012). Besides, this approach can reveal information on specific distribution of the second messenger. It has already been shown that the activation of the *cdrA* promoter with c-di-GMP takes place by releasing the repression exerted via the FleQ transcriptional regulator (Borlee et al., 2010). We have confirmed this (not shown) and the *fleQ* mutant was excluded from this analysis. Different patterns of fluorescence were observed for both mutants in the arginine biosynthetic pathways when pMAMV1 was transferred to these strains, which indicates an unequal distribution of free c-di-GMP in the streaks of these two strains. Differences were also detected between the two *argG* variants, being in one of them fluorescence detectable at the edge of the streak (**Figure 4A**). In addition, the higher intensity of fluorescence observed for *argH* compared to *argG* (**Figure 4A**) was in accordance with the c-di-GMP levels measured for these strains using the analytical method (**Figure 3**). Interestingly, the non-flocculating variant of cfcK-16 harboring pMAMV1 showed somewhat comparable fluorescence intensity to the flocculating variant, in spite of the noticeable differences in morphology observed in the streaks (**Figure 4A**), which is in accordance with the second messenger being detectable by the analytical method in both strains (**Figure 3**). Strain cfcK-16 cured of plasmid pMAMV1 presented the highest c-di-GMP levels among all the plasmid-cured strains tested (**Figure 4B**) and as we have shown above this cannot be explained as a consequence of an increase of *rup4959* expression in this genetic background.

### A Mutation in the Predicted Phosphorylation Site of Rup4959 Abrogates its Diguanylate Cyclase Activity

**FIGURE 5:**
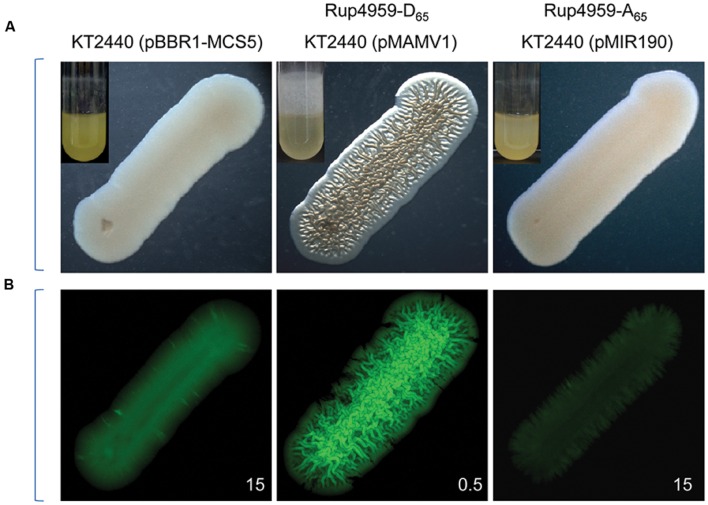
**Residue D_65_ of the REC domain of Rup4959 is essential for its diguanylate cyclase activity.** **(A)** Morphology and biofilm (inserts) of *P. putida* harboring pMAMV1 or pMIR190. Images were taken as shown in the legend for **Figure 1**. **(B)** All the indicated bacterial strains harbored pCdrA. Images of the dark fields were taken as indicated in the legend for **Figure 4**.

Unlike what was observed with the wild type, we have shown above that the global levels of c-di-GMP were undetectable in the mutant cfcK-54 carrying pMAMV1 and also that this mutant is hampered in a HK. An “*in silico*” analysis of Rup4959, which is responsible for the c-di-GMP increase that occurs in the presence of pMAMV1, predicts phosphorylation of this orphan response regulator at residue D65 of its REC domain (**Supplementary Figure S6**). To unequivocally demonstrate that this phosphorylation is required for Rup4959 to be an active DGC, the D65 was replaced with A65 by directed mutagenesis of plasmid pMAMV1 as described in Section “Materials and Methods”. It was confirmed that unlike pMAMV1, the plasmid pMIR190 encoding the mutant Rup4959-Ala65 did not confer increased biofilm or crinkle morphology (**Figure 5A**) typical of high c-di-GMP levels to KT2440. The c-di-GMP content of KT2440 overexpressing Rup4959-Ala65 detected by the biosensor was comparable to that of the wild type containing physiological content of Rup4959 (**Figure 5B**). Thus the accumulation of Rup4959-Ala65 was not enough for this protein to exhibit DGC activity indicating that the response regulator Rup4959 requires phosphorylation for its functionality.

### Autophosphorylation of His496 Residue of PP_3761, the Open Reading Frame Inactivated in cfcK-54, Is Required for the Diguanylate Cyclase Activity of Rup4959

**FIGURE 6:**
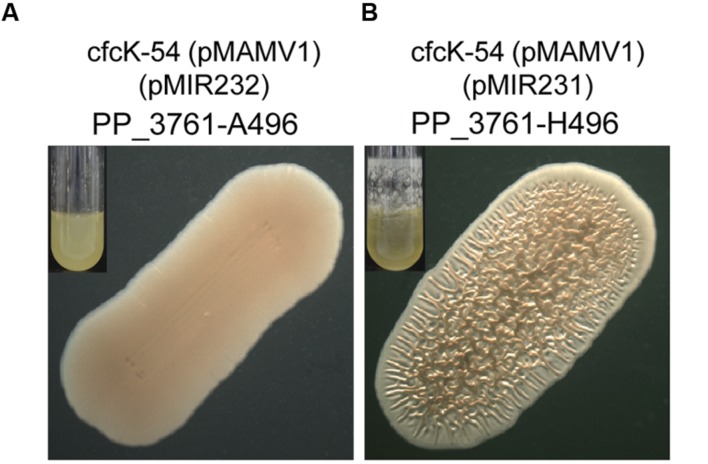
**Conserved residue H496 of the phosphoaceptor domain of PP_3761 is essential for the diguanylate cyclase activity of Rup4959.** **(A)** Phenotype of cfcK-54 expressing the variant PP_3761-A496 from pMIR232. **(B)** Phenotype of cfcK-54 expressing the wild type PP_3761-H496 from pMIR231. In both panels Rup4959 overexpressing conditions were imposed with pMAMV1; small inserts show biofilms formed after 24 h under rotation (40 rpm).

As we have shown above, mutant cfcK-54 is knocked out in PP_3761 (**Table 2**). This *orf* encodes a hybrid sensor HK, whose phosphoacceptor domain presents a conserved histidine residue (H496) which is usually *trans*-autophosphorylated by the catalytic domain present in the HK (Tomomori et al., 1999) (**Supplementary Figure S7**). To unequivocally demonstrate that this residue was required for Rup4959 to catalyze the transfer of the phosphoryl group to an aspartate residue on its receiver domain, the H496 was replaced with A496 by directed mutagenesis of plasmid pMIR231 as described in Section “Materials and Methods”. It was confirmed that unlike pMIR231 encoding the wild type HK (PP_3761-H496), the plasmid pMIR232 encoding the mutant PP_3761-A496 did not restore any of the multiple phenotypes (crinkle morphology, increased biofilm and flocculation) typical of high c-di-GMP levels to KT2440 under Rup4949 overexpression conditions (**Figure 6**). Thus the conserved autophosphorylation histidine residue (H496) of PP_3761 is essential for Rup4959 to exhibit DGC activity.

### Characterization of Mutants Hampered in the Arginine Biosynthetic Pathway

**FIGURE 7:**
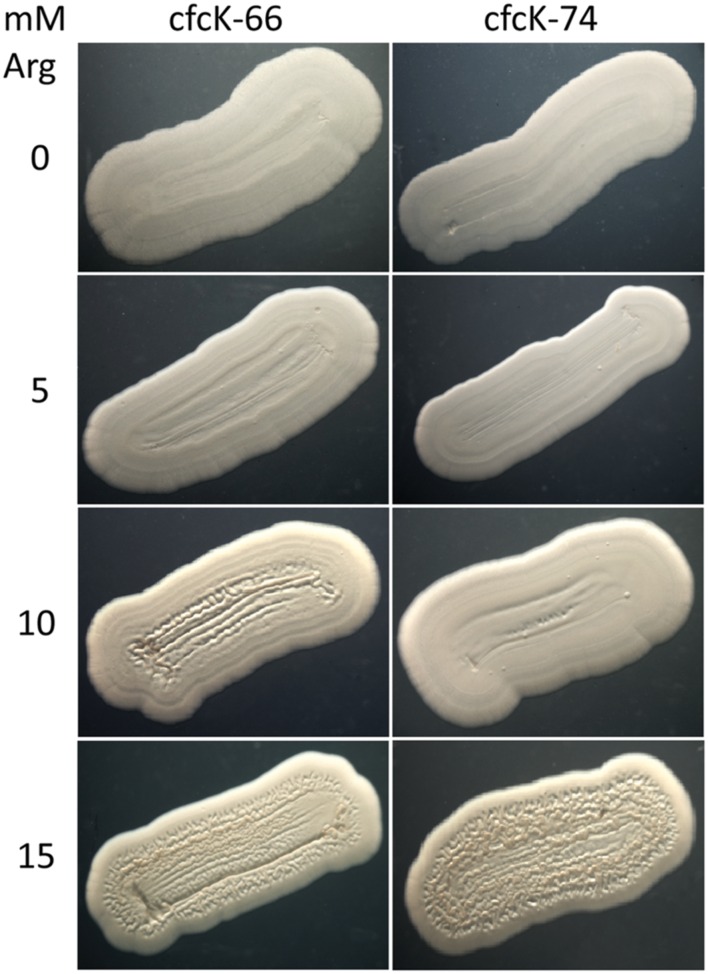
**Complementation of the cfc phenotype of *argG* and *argH* mutants by arginine.** Pictures of cfcK-66 (*argG*) and cfcK-74 (*argH*) stretches cultivated on glucose-supplemented M9 minimal medium supplied with the indicated concentrations of arginine were taken after 48 h as indicated in the legend for **Figure 1**.

It has been shown above that mutants cfcK-66 and cfcK-74 had a non-functional arginine biosynthesis pathway. As expected these mutants were confirmed to be auxotrophs for this amino acid (**Supplementary Figure S3A**). In addition, they showed a characteristic “whiter and drier” phenotype (**Figure 1**) and this characteristic was independent of carrying any plasmid. Auxotrophy was overcome by supplementing M9-glucose with arginine (1 mM) (**Supplementary Figure S3B**). Remarkably, an intact *argG* allele supplied via pME1088 only complemented the Arg auxotrophy of cfcK-66 when this strain harbored pMAMV1, even though to our knowledge there is no previous evidence that arginine biosynthesis is regulated by c-di-GMP. The presence of pMAMV1 was not a requirement in order to complement the auxotrophy of cfcK-74 with an intact *argH* allele provided via pME184. The cfc phenotype of mutants *argG* and *argH* when carrying pMAMV1 was also complemented by adding arginine, but this complementation required higher concentration of the amino acid (**Figure 7**).

Curiously, LB-agar plates of cfcK-74 showed a brownish pigment (**Supplementary Figure S8A**), a characteristic that was prevented by supplementing LB with Arg (**Supplementary Figure S9A**). This color development was not observed for cfcK-66 (**Supplementary Figure S8B**), initially suggesting that the accumulation of L-arginosuccinate might be involved in the color formation observed for cfcK-74. However, when cfcK-66 harbored pMAMV1, the development of a similar color was also observed (**Supplementary Figure S8B**). An intact *argG* almost completely prevented the color development in cfcK-66 (pMAMV1) (**Supplementary Figure S8B**). Similarly an intact *argH* allele abolished color development in cfcK-74 (**Supplementary Figure S9B**) and cfcK-74 (pMAMV1) (**Supplementary Figure S8A**). Thus plasmids pME1088 and pME184 were able to complement all phenotypes observed in these strains, including, the cfc phenotype, and the two other phenotypes associated with *argG* and *argH* mutations, namely, auxotrophy and brownish color development.

### Inverse Modulation of Free c-di-GMP Bacterial Content by Arginine and Aspartic Acid

**FIGURE 8:**
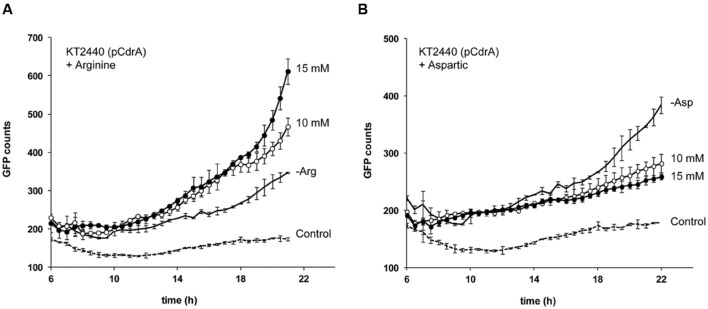
**Inverse modulation of c-di-GMP cell content by arginine and aspartic acid.** GFP counts indicate the read fluorescence corrected by the growth in LB 1/10 (OD_600 nm_). **(A)** Effect of arginine. **(B)** Effect of aspartic acid. In both panels solid lines without symbol correspond to *P. putida* KT2440 (pCdrA::*gfp*^C^); empty and solid symbols correspond to the same strain when media was supplied with 10 and 15 mM, respectively, of the amino acid; discontinued lines correspond to the background fluorescence counts detected for *P. putida* KT2440 without the reporter plasmid (Control). Average of two biological replicates, with three experimental replicates, and standard deviation are plotted.

Dissimilarities described above between cfcK-66 and cfcK-74 stimulated our interest to examine the effect that metabolic intermediates of the arginine biosynthetic pathway might have on the levels of c-di-GMP in the wild type *P. putida* strain. Since quantifying the second messenger in the wild type is not possible by analytic methods because of the low levels, we took advantage of the reporter plasmid pCdrA::*gfp*^C^. Whereas increasing concentrations of arginine up to 15 mM raised the levels of the second messenger (**Figure 8A**), aspartic acid had the opposite effect (**Figure 8B**), as an indication that the expected accumulation of the latter amino acid in the mutant cfcK-66 (*argG*) (**Supplementary Figure S2**) might be the reason for the lower c-di-GMP content observed for this strain compared to cfcK-74 (**Figures 3** and **4**).

## Discussion

In the current work we have begun to unveil the complexities of the regulatory and metabolic network associated with the overexpression of the response regulator with DGC activity Rup4959 in *P. putida.* The variety of functions identified in this work, using mutants that completely or partially lost a crinkle morphology characteristic of the increased c-di-GMP levels gives an idea of the global influence of this compound as intracellular second messenger in this bacterium. The fact that none of the genetic determinants identified here were among those previously found to be required by *P. putida* to attain a pleiotropic phenotype, including crinkle morphology (Matilla et al., 2011), suggests that additional functions may still remain uncovered. Curiously, all of the mutants identified in the current work with enhanced crinkle morphology showed the transposon to be inserted in the adhesin LapA and no further efforts were committed to identify determinants with a negative influence upon crinkle morphology.

Most of the crinkle-free colony mutants isolated did not flocculate, while some retained the ability to form somewhat increased biofilms. A relevant conclusion from the results is that, although all the phenotypic alterations in the wild type are caused by high levels of c-di-GMP, it seems that not all of these alterations are linked to each other. Thus, for example, mutants *argG* and *argH* lost the crinkly colony morphology but were still able to form a thin biofilm and flocculate in liquid medium. In addition *dnaK* and *fleQ* mutants, although unable to flocculate, still conserved and in fact showed an increased capacity of biofilm formation. Given that crinkle morphology relied on pMAMV1 and pMIR178, to ensure the consistence of the crinkle-free isolates, they were cured of their respective plasmids and their phenotypes reassessed in a minimum of 50 independent newly raised exconjugants. Except for *algU* and *argG*, no differences among clones were found for cfc mutants; heterogeneity was observed among different clones of the same strain after complementation with these two wild type alleles.

Remarkably five cfc mutants were knock-out in genes that encode proteins containing an AAA+ ATPase domain. One of them, the flagellar biosynthesis regulator FlhF, is a GTP-binding protein (Pandza et al., 2000). Interestingly another putative GTP-binding protein has been identified in this work (PP_0015) and further work must be performed to confirm whether these proteins directly interact with c-di-GMP. Additional AAA+ ATPase domain proteins identified were the flagellum-specific ATP synthase FliI, which is a component of the flagellar export apparatus and whose interaction with c-di-GMP has been recently reported (Trampari et al., 2015), and the ATP-binding component MetN1 of a methionine import transporter. We currently cannot explain why MetN1 is essential to acquire crinkle phenotype, but it suggests that import ATPases may also be allosterically regulated by c-di-GMP. Finally, the identification of the AAA+ ATPase domain containing protein FleQ in this work was not unexpected. This transcriptional regulator involved in flagellar gene expression has been shown to modulate the expression of certain EPSs of *P. aeruginosa* in a c-di-GMP dependent manner. In this organism, FleQ binds the second messenger (Baraquet and Harwood, 2013), and this binding allows activation of the expression of the *pel* operon (Baraquet et al., 2012). We have confirmed that in *P. putida* KT2440 FleQ also binds c-di-GMP and interacts with the regulatory regions of EPSs biosynthesis (our unpublished results). In this bacterium, four gene clusters code for the production of cellulose, alginate and two species-specific EPSs named Pea and Peb (Nelson et al., 2002; Nilsson et al., 2011). Given that the cluster of genes encoding Pea was essential for the development of crinkle morphology under augmented c-di-GMP (Matilla et al., 2011), we hypothesized that FleQ might participate in Pea biosynthesis and direct interaction between FleQ and the regulatory region of *pea* has been recently documented (our unpublished results). In addition, the transcription of *lapA* was compromised in a *fleQ* mutant (Martínez-Gil et al., 2014). LapA is the major determinant of biofilm formation, and this can explain why the other characteristics related to increased c-di-GMP that were tested in this study, including flocculation and biofilm formation capacity, were abolished or diminished in this mutant. In addition to the role that FleQ might exert in KT2440 as a consequence of binding c-di-GMP, we confirmed that the expression of *rup4959* was notably reduced in the *fleQ* background during the stationary phase of growth. Our “*in vitro*” preliminary evidences suggest that this repression is exerted indirectly although “*in vivo*” ChIP-sequencing experiments with FleQ are currently being performed and will shed light on this issue.

A second transcriptional regulator identified in our screen is the alternative sigma factor AlgU which is also involved in EPS synthesis in *P. aeruginosa*. In *P. aeruginosa* AlgU, in coordination with the anti-sigma factor MucA, controls the expression of the alginate synthesis operon leading to a mucoid phenotype (Yin et al., 2013) and regulation of biofilm formation (Bazire et al., 2010). In *P. fluorescens*, a regulatory cascade controlling flagellar motility has been proposed, in which the two-component system GacS/GacA indirectly controls expression of AlgU, which in turn acts as an indirect inhibitor of expression of *fleQ* by activating its repressor AmrZ (Martínez-Granero et al., 2012). In this work several crinkle-free mutants with a transposon inserted at different positions in the last codon of *algU* were isolated. Given the absence of any observed mutant phenotype in a knock-out *algU* strain (not shown), it is tempting to speculate that the transposon insertion at the end of *algU* in cfcK-16 had caused a polar effect upon the expression of *mucA*, which encodes the AlgU anti-sigma factor MucA (Schurr et al., 1996). We are currently investigating the possibility that an altered expression of MucA took place in this strain and as a consequence an increase in the AlgU/MucA ratio was attained. This might explain the cfc phenotype of this variant.

The two-component regulatory system GacS/GacA was also identified in this work. This system regulates social behavior, expression of virulence factors and the synthesis of antimicrobial secondary metabolites in different bacteria (Heeb and Haas, 2001; Lapouge et al., 2008). In response to an as-yet unidentified environmental signal, GacS activates the transcriptional regulator GacA, which subsequently triggers expression of small RNAs. This results in a regulatory cascade often leading to the translation of target genes being de-repressed, with the participation of proteins of the CsrA (carbon storage regulator) family. Putative CsrA-binding sites were identified in the mRNA leader sequence of *rup4959* (Matilla et al., 2011), whose effects upon gene expression and thus the regulation by components of this family of proteins in KT2440 are currently being analyzed (our unpublished results). In addition to this post-transcriptional regulation that eventually relies upon GacA, the transcription of *rup4959* is RpoS dependent and the two component system GacS/GacA is essential for the expression of *rpoS* in KT2440 (Martínez-Gil et al., 2014). Interestingly the chaperone encoded by *dnaK*, also identified in this work, was found to be positively regulated by GacA/GacS in another *P. putida* strain (Dubern et al., 2005). Whether DnaK might affect the pattern of EPS biosynthesis and as a consequence almost eradicate the crinkle phenotype is unknown. It is known that a mutation in *dnaK* of KT2440 causes accumulation of heat shock proteins (Kobayashi et al., 2011). One can speculate that protein misfolding arises because of enhanced c-di-GMP, in which case chaperones might be essential to develop crinkle morphology.

**FIGURE 9:**
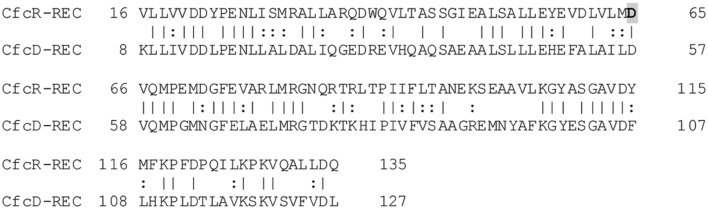
**Alignment of CfcR and CfcD REC domains.** Domains were defined as described in the MIST database (Ulrich and Zhulin, 2010) and aligned using EMBOSS Needle (Rice et al., 2000). Phosphorylated D65 in CfcR is highlighted in gray.

The paradigmatic response regulator PleD requires phosphorylation-mediated dimerization for its functionality as a DGC (Paul et al., 2007). The response regulator Rup4959 is predicted to be phospohrylated at position D65 (**Supplementary Figure S6**). At the beginning of this work it was unknown whether such post-translational modification was a requisite for the activity exhibited by Rup4959. Given that the D65 and other conserved residues at its receiver domain suggested so, we decided to substitute D65 with A65 and have concluded that an aspartic acid residue at this position is required for the functionality of this response regulator as a DGC. Interestingly a sensor HK was identified in this work as being essential for crinkle morphology. The locus encoding this HK is the first *orf* of a cluster of five genes that we propose to name *cfcABCDE*. The genes *cfcC* and *cfcB* encode one of the three chemosensing systems CheB/CheR present in *P. putida*, although the role of CfcB and CfcC in chemotaxis has been ruled out (García-Fontana et al., 2013). Because mini-Tn5[Km1] often causes non-polar mutations (Ramos, personal communication), it was likely that the cfc phenotype was due to the inactivation of *cfcA*. To unequivocally demonstrate that the phenotype exhibited by the mutant cfcK-54 was due to the inactivation *cfcA* we have complemented the cfc phenotype by ectopic expression of this gene. In addition we have confirmed that a *cfcA* null mutant generated by allelic replacement shares the same phenotype as cfcK-54 and mutants in the other genes of the *cfc* cluster are not hampered in crinkle phenotype (our unpublished results). Thus we propose CfcA as the HK most likely transferring the phosphoryl group to the D65 residue on the receiver domain of Rup4959. In support of this, a point mutation in the autophosphorylation H496 of CfcA caused loss of activity in Rup4959. Furthermore, we identified that the REC domain present in CfcD showed the highest score (corresponding to 48% identity and 66% similarity) found in KT2440 to the REC domain of Rup4959 (**Figure 9**), suggesting that both domains are candidates to be phosphorylated by the same HK, namely CfcA. Genes encoding sensor kinase/response regulator systems often appear clustered in the genome. Remarkably genes orthologous to *cfcA* and *rup4959* come together in the chromosome of *P. resinovorans* NBRC 106553 (Winsor et al., 2011), suggesting they are functionally related. With these genetic evidences, we have renamed the response regulator Rup4959 as CfcR.

Another block of mutants identified are related to metabolic processes. We have focused our attention particularly on two genes in the arginine biosynthetic pathway, *argG* and *argH*. Interestingly other mutants in the biosynthetic pathways of this amino acid, such as *argA* and *argB* did not lose their crinkle morphology when carrying pMAMV1 (Molina, personal communication), in spite of being auxotrophic for arginine, suggesting that auxotrophy for this amino acid may not be the only or ultimate cause for the crinkle-free morphology exhibited by *argG* and *argH* mutants. At this point the connection between this metabolic pathway and c-di-GMP in *Pseudomonas* is unclear. However, it is worth mentioning that previous reports indicate a connection between amino acids and bacterial social behaviors. D-amino acids have been shown to promote dispersal of *Bacillus subtilis* biofilms (Kolodkin-Gal et al., 2010). On the other hand, several L-amino acids reduce swarming motility of *P. aeruginosa* and promote biofilm formation when added exogenously to the growth medium; of these, arginine at a concentration of 4.8 mM completely inhibited swarming (Bernier et al., 2011). Although it was not the most prominent in terms of biofilm promotion, L-arginine supplementation resulted in a 1.7-fold increase in c-di-GMP content of *P. aeruginosa* cells compared to the content of cells grown solely on glucose. More recently, exogenous L-arginine has been found to induce a rapid translation independent increase in c-di-GMP concentrations in *Salmonella* Typhimurium. This response requires the arginine transporter subunit ArtI and a DGC containing a periplasmic Arg-sensing domain (Mills et al., 2015). Based on the c-di-GMP biosensor pCdrA (Rybtke et al., 2012), our data obtained with *argG* and *argH* mutants suggest that an intact arginine biosynthetic pathway positively influences free c-di-GMP content (**Figure 4B**), although *cfcR* transcription was not altered in these mutants (our unpublished results). In fact if, as it is presumable, accumulation of aspartic acid takes place in a mutant *argG*, we hypothesized that this amino acid might exert a negative effect upon the free content of the second messenger in the bacteria cell. We could confirm this hypothesis (**Figure 8**) although the underlying mechanism remains unknown. Given that species specific EPS Pea and LPS were known to be essential for *P. putida* to acquire the crinkle phenotype (Matilla et al., 2011), it is possible that the transduction of c-di-GMP into biopolymer production pathways is compromised in the *argH* mutant. However, the *argG* and *argH* mutant strains showed increased biofilm formation capacity and a full flocculation phenotype under *cfcR* overexpression conditions, indicating that the adhesins LapA and LapF, the major determinants of biofilm formation (Hinsa et al., 2003; Martínez-Gil et al., 2010) and flocculation (our unpublished results), were still active in these mutants. As we have seen above, the phenotypes of these strains differed markedly from that of the *fleQ* mutant, in which the transcription of *lapA* was compromised (Martínez-Gil et al., 2014).

In *P. putida* there is no clear equivalent of the above mentioned DGC STM1987 containing a periplasmic Arg-sensing domain, what emphasizes the interest in unveiling the mechanism by which arginine may control c-di-GMP levels in this bacterium and related species. We are currently investigating the specific role of CfcA/CfcR in the modulation of c-di-GMP content by metabolites of the arginine biosynthesis pathway. In addition we are examining the different steps of this pathway to determine their influence on c-di-GMP global cell content and their concomitant effect on signal transduction.

## Author Contributions

MIR-G and ME-U conceived the study. MIR-G designed the research. MLT and MIS performed molecular work. MIR-G, MLT and LB-M took pictures. MAM organized and performed the sampling and analyzed the data for c-di-GMP quantification. OH-R performed experiments with TECAN. OH-R, LB-M, and VGT performed biofilm experiments and helped with data interpretation. MIR-G and ME-U wrote the manuscript and discussed the results and all the authors specially MAM commented on the manuscript.

## Conflict of Interest Statement

The authors declare that the research was conducted in the absence of any commercial or financial relationships that could be construed as a potential conflict of interest.

The handling Editor declared a shared affiliation, though no other collaboration with all the authors and states that the process nevertheless met the standards of a fair and objective review.

## References

[B1] Baraquet C. Harwood C. S. (2013). Cyclic diguanosine monophosphate represses bacterial flagella synthesis by interacting with the Walker A motif of the enhancer-binding protein FleQ. *Proc. Natl. Acad. Sci. U.S.A.* 110 18478–18483. 10.1073/pnas.1318972110 24167275 PMC3832005

[B2] Baraquet C. Murakami K. Parsek M. R. Harwood C. S. (2012). The FleQ protein from *Pseudomonas aeruginosa* functions as both a repressor and an activator to control gene expression from the pel operon promoter in response to c-di-GMP. *Nucleic Acids Res.* 40 7207–7218. 10.1093/nar/gks384 22581773 PMC3424551

[B3] Bazire A. Shioya K. Soum-Soutéra E. Bouffartigues E. Ryder C. Guentas-Dombrowsky L. (2010). The sigma factor AlgU plays a key role in formation of robust biofilms by nonmucoid *Pseudomonas aeruginosa*. *J. Bacteriol.* 192 3001–3010. 10.1128/JB.01633-09 20348252 PMC2901682

[B4] Bernier S. P. Ha D. G. Khan W. Merritt J. H. O’Toole G. A. (2011). Modulation of *Pseudomonas aeruginosa* surface-associated group behaviors by individual amino acids through c-di-GMP signaling. *Res. Microbiol.* 162 680–688. 10.1016/j.resmic.2011.04.014 21554951 PMC3716369

[B5] Bertani G. (1951). Studies on lysogenesis. I. The mode of phage liberation by lysogenic *Escherichia coli. J. Bacteriol.* 62 293–300. 14888646 10.1128/jb.62.3.293-300.1951 PMC386127

[B6] Borlee B. R. Goldman A. D. Murakami K. Samudrala R. Wozniak D. J. Parsek M. R. (2010). *Pseudomonas aeruginosa* use a cyclic-di-GMP-regulated adhesin to reinforce the biofilm extracellular matrix. *Mol. Microbiol.* 75 827–842. 10.1111/j.1365-2958.2009.06991.x 20088866 PMC2847200

[B7] Boyd C. D. O’Toole G. A. (2012). Second messenger regulation of biofilm formation: breakthroughs in understanding c-di-GMP effector systems. *Annu. Rev. Cell Dev. Biol.* 28 439–462. 10.1146/annurev-cellbio-101011-155705 23057745 PMC4936406

[B8] Choi K. H. Kumar A. Schweizer H. P. (2006). A 10-min method for preparation of highly electrocompetent *Pseudomonas aeruginosa* cells: application for DNA fragment transfer between chromosomes and plasmid transformation. *J. Microbiol. Methods* 64 391–397. 10.1016/j.mimet.2005.06.001 15987659

[B9] DeLorenzo V. Herrero M. Jakubzik U. Timmis K. N. (1990). Mini-Tn5 transposon derivatives for insertion mutagenesis, promoter probing, and chromosomal insertion of cloned DNA in gram-negative eubacteria. *J. Bacteriol.* 172 6568–6572. 2172217 10.1128/jb.172.11.6568-6572.1990 PMC526846

[B10] Dubern J. F. Lagendijk E. L. Lugtenberg B. J. J. Bloemberg G. V. (2005). The heat shock genes dnaK, dnaJ, and grpE are involved in regulation of putisolvin biosynthesis in *Pseudomonas putida* PCL1445. *J. Bacteriol.* 187 5967–5976. 10.1128/JB.187.17.5967-5976.2005 16109938 PMC1196155

[B11] Espinosa-Urgel M. Ramos J. L. (2004). Cell density dependent gene contributes to efficient seed colonization by *Pseudomonas putida* KT2440. *Appl. Environ. Microbiol.* 70 5190–5198. 10.1128/AEM.70.9.5190-5198.2004 15345399 PMC520864

[B12] Fürste J. P. Pansegrau W. Frank R. Blocker H. Scholz P. Bagdasarian M. (1986). Molecular cloning of the plasmid RP4 primase region in a multi-host-range tacP expression vector. *Gene* 48 119–131. 10.1016/0378-1119(86)90358-6 3549457

[B13] Galperin M. Y. (2006). Structural classification of bacterial response regulators: diversity of output domains and domain combinations. *J. Bacteriol.* 188 4169–4182. 10.1128/JB.01887-05 16740923 PMC1482966

[B14] García-Fontana C. Reyes-Darias J. A. Muñoz-Martínez F. Alfonso C. Morel B. Ramos J. L. (2013). High specificity in CheR methyltransferase function: CheR2 of *Pseudomonas putida* is essential for chemotaxis, whereas CheR1 is involved in biofilm formation. *J. Biol. Chem.* 288 18987–18999. 10.1074/jbc.M113.472605 23677992 PMC3696673

[B15] Heeb S. Haas D. (2001). Regulatory roles of the GacS/GacA two-component system in plant-associated and other Gram-negative bacteria. *Mol. Plant Microbe Interact.* 14 1351–1363. 10.1094/MPMI.2001.14.12.1351 11768529

[B16] Hengge R. (2009). Principles of c-di-GMP signalling in bacteria. *Nat. Rev. Microbiol.* 7 263–273. 10.1038/nrmicro2109 19287449

[B17] Henry J. T. Crosson S. (2011). Ligand-binding PAS domains in a genomic, cellular, and structural context. *Annu. Rev. Microbiol.* 65 261–286. 10.1146/annurev-micro-121809-151631 21663441 PMC3298442

[B18] Hickman J. W. Harwood C. S. (2008). Identification of FleQ from *Pseudomonas aeruginosa* as a c-di-GMP-responsive transcription factor. *Mol. Microbiol.* 69 376–389. 10.1111/j.1365-2958.2008.06281.x 18485075 PMC2612001

[B19] Hinsa S. M. Espinosa-Urgel M. Ramos J. L. O’Toole G. A. (2003). Transition from reversible to irreversible attachment during biofilm formation by *Pseudomonas fluorescens* WCS365 requires an ABC transporter and a large secreted protein. *Mol. Microbiol.* 49 905–918. 10.1046/j.1365-2958.2003.03615.x 12890017

[B20] Huang B. Whitchurch C. B. Mattick J. S. (2003). FimX, a multidomain protein connecting environmental signals to twitching motility in *Pseudomonas aeruginosa*. *J. Bacteriol.* 185 7068–7076. 10.1128/JB.185.24.7068-7076.2003 14645265 PMC296245

[B21] Kazmierczak B. I. Lebron M. B. Murray T. S. (2006). Analysis of FimX, a phosphodiesterase that governs twitching motility in *Pseudomonas aeruginosa*. *Mol. Microbiol.* 60 1026–1043. 10.1111/j.1365-2958.2006.05156.x 16677312 PMC3609419

[B22] Kobayashi Y. Ohtsu I. Fujimura M. Fukumori F. (2011). A mutation in dnaK causes stabilization of the heat shock sigma factor σ32, accumulation of heat shock proteins and increase in toluene-resistance in *Pseudomonas putida*. *Environ. Microbiol.* 13 2007–2017. 10.1111/j.1462-2920.2010.02344.x 20880327

[B23] Kolodkin-Gal I. Romero D. Cao S. Clardy J. Kolter R. Losick R. (2010). D-amino acids trigger biofilm disassembly. *Science* 328 627–629. 10.1126/science.1188628 20431016 PMC2921573

[B24] Kovach M. E. Elzer P. H. Hill D. S. Robertson G. T. Farris M. A. Roop R. M. II (1995). Four new derivatives of the broad-host-range cloning vector pBBR1MCS, carrying different antibiotic-resistance cassettes. *Gene* 166 175–176. 10.1016/0378-1119(95)00584-1 8529885

[B25] Lapouge K. Schubert M. Allain F. H. Haas D. (2008). Gac/Rsm signal transduction pathway of gamma-proteobacteria: from RNA recognition to regulation of social behaviour. *Mol. Microbiol.* 67 241–253. 10.1111/j.1365-2958.2007.06042.x 18047567

[B26] Martínez-Gil M. Ramos-González M. I. Espinosa-Urgel M. (2014). Roles of cyclic Di-GMP and the Gac system in transcriptional control of the genes coding for the *Pseudomonas putida* adhesins LapA and LapF. *J. Bacteriol.* 196 1484–1495. 10.1128/JB.01287-13 24488315 PMC3993364

[B27] Martínez-Gil M. Yousef-Coronado F. Espinosa-Urgel M. (2010). LapF, the second largest *Pseudomonas putida* protein, contributes to plant root colonization and determines biofilm architecture. *Mol. Microbiol.* 77 549–561. 10.1111/j.1365-2958.2010.07249.x 20545856

[B28] Martínez-Granero F. Navazo A. Barahona E. Redondo-Nieto M. Rivilla R. Martín M. (2012). The Gac-Rsm and SadB signal transduction pathways converge on AlgU to downregulate motility in *Pseudomonas fluorescens*. *PLoS ONE* 7:e31765 10.1371/journal.pone.0031765 PMC3282751 22363726

[B29] Matilla M. A. Espinosa-Urgel M. Rodríguez-Herva J. J. Ramos J. L. Ramos-González M. I. (2007). Genomic analysis reveals the major driving forces of bacterial life in the rhizosphere. *Genome Biol.* 8:R179 10.1186/gb-2007-8-9-r179 PMC2375017 17784941

[B30] Matilla M. A. Travieso M. L. Ramos J. L. Ramos-González M. I. (2011). Cyclic diguanylate turnover mediated by the sole GGDEF/EAL response regulator in *Pseudomonas putida*: its role in the rhizosphere and an analysis of its target processes. *Environ. Microbiol.* 13 1745–1766. 10.1111/j.1462-2920.2011.02499.x 21554519

[B31] Mills E. Petersen E. Kulasekara B. R. Miller S. I. (2015). A direct screen for c-di-GMP modulators reveals a *Salmonella Typhimurium* periplasmic L-arginine-sensing pathway. *Sci. Signal.* 8:ra57 10.1126/scisignal.aaa1796 26060330

[B32] Nelson K. E. Weinel C. Paulsen I. T. Dodson R. J. Hilbert H. Martins dos Santos V. A. P. (2002). Complete genome sequence and comparative analysis of the metabolically versatile *Pseudomonas putida* KT2440. *Environ. Microbiol.* 4 799–808. 10.1046/j.1462-2920.2002.00366.x 12534463

[B33] Nilsson M. Chiang W. C. Fazli M. Gjermansen M. Givskov M. Tolker-Nielsen T. (2011). Influence of putative exopolysaccharide genes on *Pseudomonas putida* KT2440 biofilm stability. *Environ. Microbiol.* 13 1357–1369. 10.1111/j.1462-2920.2011.02447.x 21507178

[B34] O’Toole G. A. Kolter R. (1998). Initiation of biofilm formation in *Pseudomonas fluorescens* WCS365 proceeds via multiple, convergent signalling pathways: a genetic analysis. *Mol. Microbiol.* 28 449–461. 10.1046/j.1365-2958.1998.00797.x 9632250

[B35] Pandza S. Baetens M. Park C. H. Au T. Keyhan M. Matin A. (2000). The G-protein FIhF has a role in polar flagellar placement and general stress response induction in *Pseudomonas putida*. *Mol. Microbiol.* 36 414–423. 10.1046/j.1365-2958.2000.01859.x 10792727

[B36] Paul R. Abel S. Wassmann P. Beck A. Heerklotz H. Jenal U. (2007). Activation of the diguanylate cyclase PleD by phosphorylation-mediated dimerization. *J. Biol. Chem.* 282 29170–29177. 10.1074/jbc.M704702200 17640875

[B37] Ramos-González M. I. (1993). *Obtención y caracterización de anticuerpos monoclonales contra Pseudomonas putida portadora de ADN recombinante: Clonación de un gen que determina un antígeno de superficie.* Ph.D. thesis, University of Granada, Granada, Spain.

[B38] Regenhardt D. Heuer H. Heim S. Fernandez D. U. Strömpl C. Moore E. R. B. (2002). Pedigree and taxonomic credentials of *Pseudomonas putida* strain KT2440. *Environ. Microbiol.* 4 912–915. 10.1046/j.1462-2920.2002.00368.x 12534472

[B39] Rice P. Longden I. Bleasby A. (2000). EMBOSS: the european molecular biology open software suite. *Trends. Genet.* 16 276–277. 10.1016/S0168-9525(00)02024-2 10827456

[B40] Römling U. Galperin M. Y. Gomelskyc M. (2013). Cyclic di-GMP: the first 25 years of a universal bacterial second messenger. *Microbiol. Mol. Biol. Rev.* 77 1–52. 10.1128/MMBR.00043-12 23471616 PMC3591986

[B41] Römling U. Gomelsky M. Galperin M. Y. (2005). C-di-GMP: the dawning of a novel bacterial signaling system. *Mol. Microbiol.* 57 629–639. 10.1111/j.1365-2958.2005.04697.x 16045609

[B42] Römling U. Simm R. (2009). Prevailing concepts of c-di-GMP signaling. *Contrib. Microbiol.* 16 161–181. 10.1159/000219379 19494585

[B43] Rybtke M. T. Borlee B. R. Murakami K. Irie Y. Hentzer M. Nielsen T. E. (2012). Fluorescence-based reporter for gauging cyclic di-GMP levels in *Pseudomonas aeruginosa*. *Appl. Environ. Microbiol.* 78 5060–5069. 10.1128/AEM.00414-12 22582064 PMC3416407

[B44] Sambrook J. Fritsch E. F. Maniatis T. (1989). *Molecular Cloning: a Laboratory Manual*, 2nd Edn New York, NY: Cold Spring Harbor Laboratory Press.

[B45] Schurr M. J. Yu H. Martinez-Salazar J. M. Boucher J. C. Deretic V. (1996). Control of AlgU, a member of the sigma E-like family of stress sigma factors, by the negative regulators MucA and MucB and *Pseudomonas aeruginosa* conversion to mucoid in cystic fibrosis. *J. Bacteriol.* 178 4997–5004. 8759866 10.1128/jb.178.16.4997-5004.1996 PMC178285

[B46] Simm R. Morr M. Kader A. Nimtz M. Römling U. (2004). GGDEF and EAL domains inversely regulate cyclic di-GMP levels and transition from sessility to motility. *Mol. Microbiol.* 53 1123–1134. 10.1111/j.1365-2958.2004.04206.x 15306016

[B47] Simon R. Priefer U. Puhler A. (1983). A broad host range mobilization system for in vivo genetic engineering: transposon mutagenesis in gram-negative bacteria. *Nat. Biotechnol.* 1 784–791. 10.1038/nbt1183-784

[B48] Spangler C. Böhm A. Jenal U. Seifert R. Kaever V. (2010). A liquid chromatography-coupled tandem mass spectrometry method for quantitation of cyclic di-guanosine monophosphate. *J. Microbiol. Methods* 81 226–231. 10.1016/j.mimet.2010.03.020 20385176

[B49] Tomomori C. Tanaka T. Dutta R. Park H. Saha S. K. Zhu Y. (1999). Solution structure of the homodimeric core domain of *Escherichia coli* histidine kinase EnvZ. *Nat. Struct. Biol.* 68 729–734. 10426948 10.1038/11495

[B50] Trampari E. Stevenson C. E. M. Little R. H. Wilhelm T. Lawson D. M. Malone J. G. (2015). Bacterial rotary export ATPases are allosterically regulated by the nucleotide second messenger cyclic-di-GMP. *J. Biol. Chem.* 290 24470–24483. 10.1074/jbc.M115.661439 26265469 PMC4591828

[B51] Ulrich L. E. Zhulin I. B. (2010). The MiST2 database: a comprehensive genomics resource on microbial signal transduction. *Nucleic Acids Res.* 38 D401–D407. 10.1093/nar/gkp940 19900966 PMC2808908

[B52] Wei X. Huang X. Tang L. Wu D. Xu Y. (2013). Global control of GacA in secondary metabolism, primary metabolism, secretion systems, and motility in the rhizobacterium *Pseudomonas aeruginosa* M18. *J. Bacteriol.* 195 3387–3400. 10.1128/JB.00214-13 23708134 PMC3719553

[B53] Winsor G. L. Lam D. K. Fleming L. Lo R. Whiteside M. D. Yu N. Y. (2011). *Pseudomonas* genome database: improved comparative analysis and population genomics capability for *Pseudomonas* genomes. *Nucleic Acids Res.* 39 D596–D600. 10.1093/nar/gkq869 20929876 PMC3013766

[B54] Woodcock D. M. Crowther P. J. Doherty J. Jefferson S. DeCruz E. Noyer-Weidner M. (1989). Quantitative evaluation of *Escherichia coli* host strains for tolerance to cytosine methylation in plasmid and phage recombinants. *Nucleic Acids Res.* 17 3469–3478. 10.1093/nar/17.9.3469 2657660 PMC317789

[B55] Yin Y. Damron F. H. Withers T. R. Pritchett C. L. Wang X. Schurr M. J. (2013). Expression of mucoid induction factor MucE is dependent upon the alternative sigma factor AlgU in *Pseudomonas aeruginosa*. *BMC Microbiol.* 13:232 10.1186/1471-2180-13-232 PMC3819740 24138584

[B56] Yousef-Coronado F. Travieso M. L. Espinosa-Urgel M. (2008). Different, overlapping mechanisms for colonization of abiotic and plant surfaces by *Pseudomonas putida*. *FEMS Microbiol. Lett.* 288 118–124. 10.1111/j.1574-6968.2008.01339.x 18783437

