# Supplementary material for: Genetic Dissection of the Regulatory Network Associated with High c-di-GMP Levels in Pseudomonas putida KT2440
Source: Front Microbiol. 2016 Jul 20;7:1093. doi: 10.3389/fmicb.2016.01093 (PMC4951495; doi:10.3389/fmicb.2016.01093)
Supplement: Supplementary file 1 [file Presentation_1.PDF]

Supplementary Material

Genetic Dissection of the Regulatory Network Associated with High C-di-GMP Levels in *Pseudomonas putida* KT2440

María Isabel Ramos-González*, María L. Travieso, María Isabel Soriano, Óscar Huertas-Rosales, Miguel A. Matilla, Laura Barrientos-Moreno, Víctor G. Tagua, Manuel Espinosa-Urgel

*** Correspondence:** María Isabel Ramos-González: maribel.ramos@eez.csic.es

SUPPLEMENTARY METHODS

Construction of plasmids pMIR200 and pMIR219

SUPPLEMENTARY FIGURES AND TABLES

Supplementary Figure S1
Supplementary Figure S2
Supplementary Figure S3
Supplementary Figure S4
Supplementary Figure S5
Supplementary Figure S6
Supplementary Figure S7
Supplementary Figure S8
Supplementary Figure S9
Supplementary Table S1
Supplementary Table S2

REFERENCES

SUPPLEMENTARY METHODS

Construction of plasmid pMIR200. Plasmid pMAMV1 (Matilla et al., 2011) was used as template to amplify the promoter of *rup4959* using oligos 5'GGTACCAGCGCTACCTGAAA3' and 5'GCATGCGTTATGTGACCGACTTCAA3'. The fragment was cloned at the sites KpnI and SphI of pMP220 (Spaink et al., 1987). Absence of further mutations was confirmed by sequencing.

Construction of plasmid pMIR219. Plasmid pMAMV1 was used as template to amplify a fragment with the promoter and the start codon of *rup4959* using oligos 5'GGTACCAGCGCTACCTGAAA3' and 5'AGATCTGCATCCATGCTGCTTCTC3'. The fragment was cloned at the Acc65I and BamHI sites of pMP220-BamHI (Matilla et al., 2011). Absence of further mutations was confirmed by sequencing.

SUPPLEMENTARY FIGURES

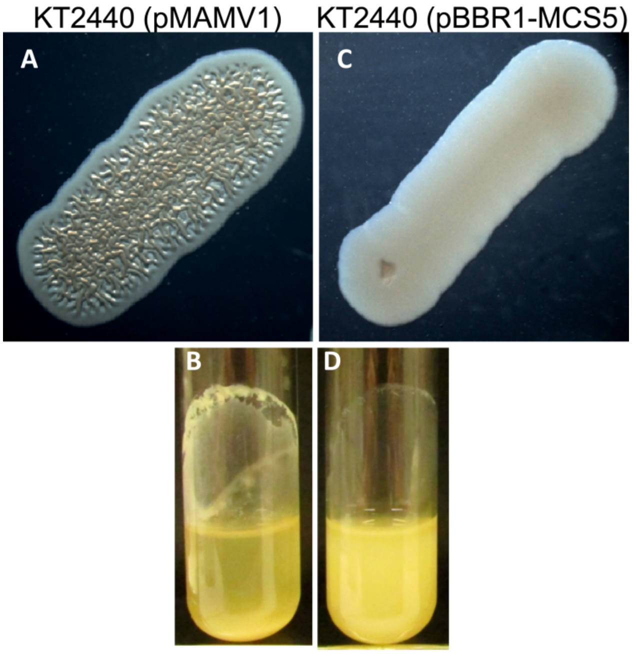

Supplementary Figure S1. Effect of overexpressing diguanylate cyclase Rup4949 from pMAMV1 upon streak morphology, biofilm formation capacity and flocculation of *Pseudomonas putida* KT2440. Plasmid pMAMV1 (A and B), containing *rup4959*, is derived from cloning vector pBBR1-MCS5 (C and D). LB-agar plates supplemented with Gm 50 µg/mL were incubated at 30°C for 48 h and pictures were taken using Leika stereomicroscope M165FC. LB cultures were incubated at 30°C for 16 h under orbital shaking (200 rpm) and pictures were taken after maintaining the tubes in static conditions for 30 minutes. As a consequence of cell flocculation on the bottom of the tube, the liquid culture appears less turbid (in panel B than in panel A).

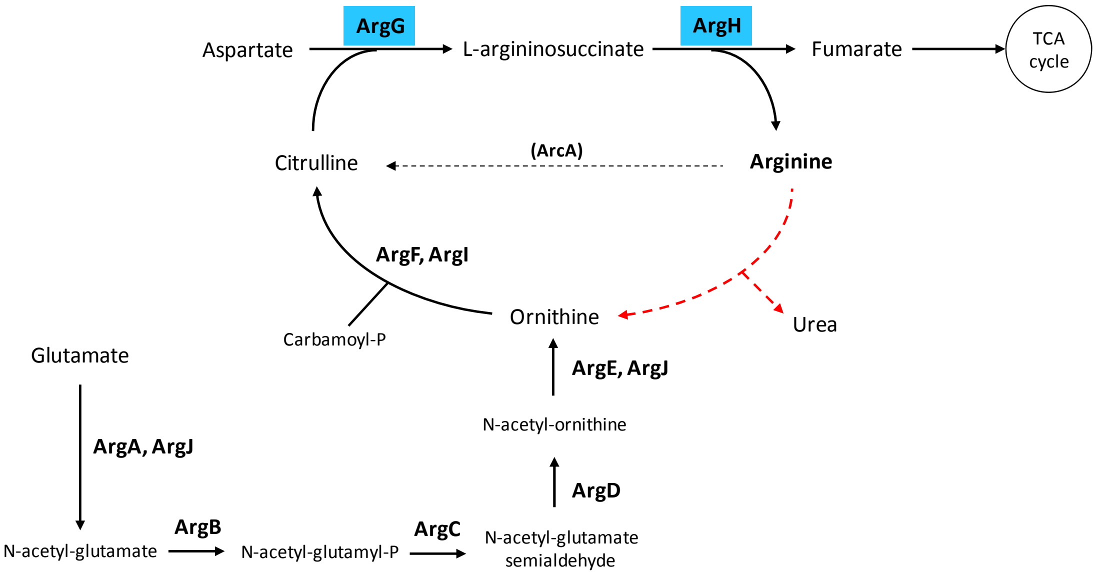

Supplementary Figure S2. Arginine biosynthetic pathway in *Pseudomonas putida* KT2440. It is derived from genetic information and data extracted from KEGG (http://www.genome.jp/kegg/). The genes identified in the cfc screen are highlighted in blue. Transcription of ArcA (broken black line) has been described to take place under anaerobic conditions in *P. aeruginosa* (Gamper et al., 1991). The step shown by the broken red line appears to be absent in KT2440.

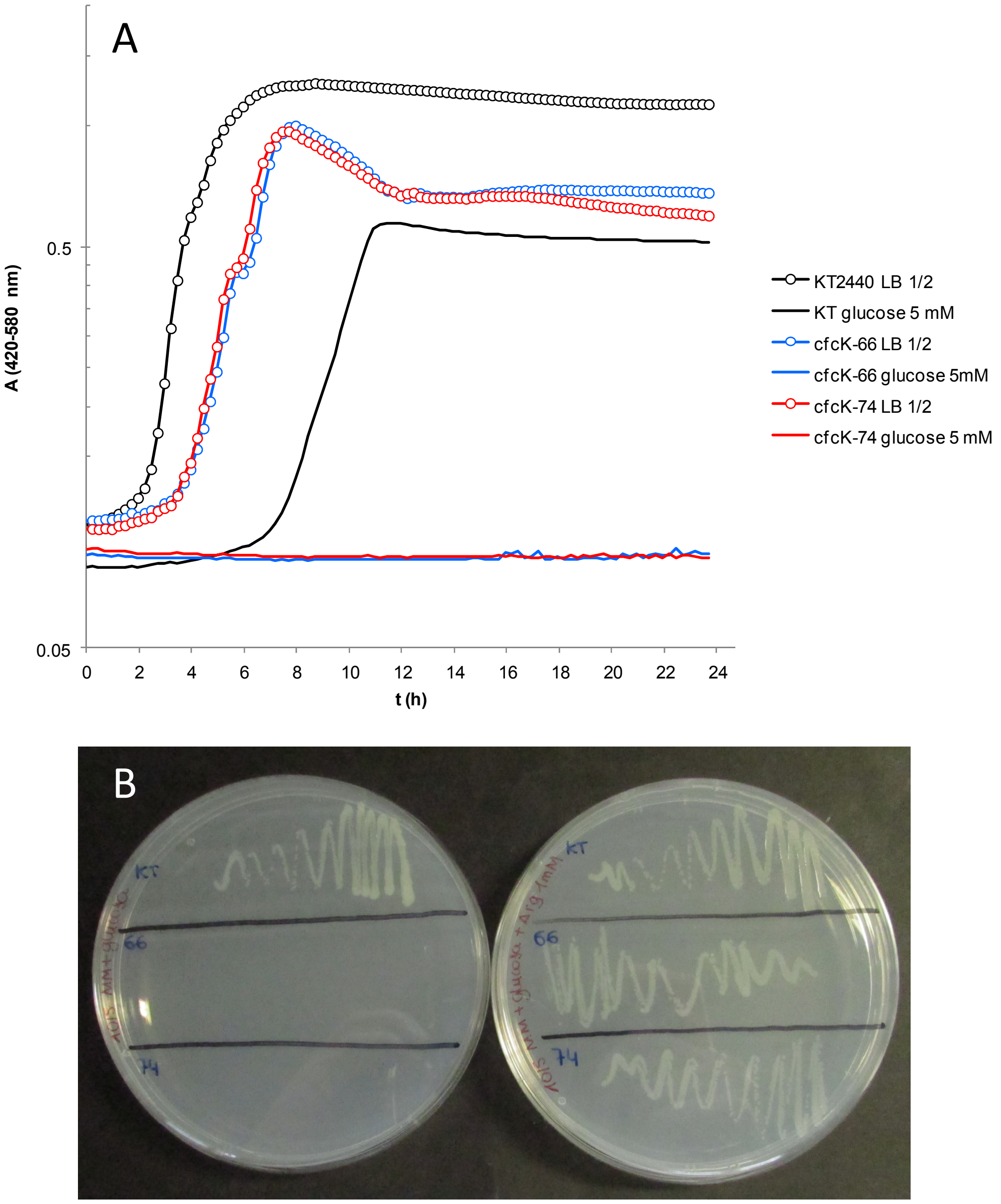

Supplementary Figure S3. **CfcK-66 and cfcK-74 strains are auxotrophic for arginine.** (A) Growth curve of cfcK-66 (*argG*) and cfcK-74 (*argH*) strains in rich and defined medium. Growth was followed automatically with a Bioscreen-C in ½ LB rich medium and M9-Glucose supplied medium (5mM). (B) Complementation of the auxotrophy by adding arginine 1 mM.

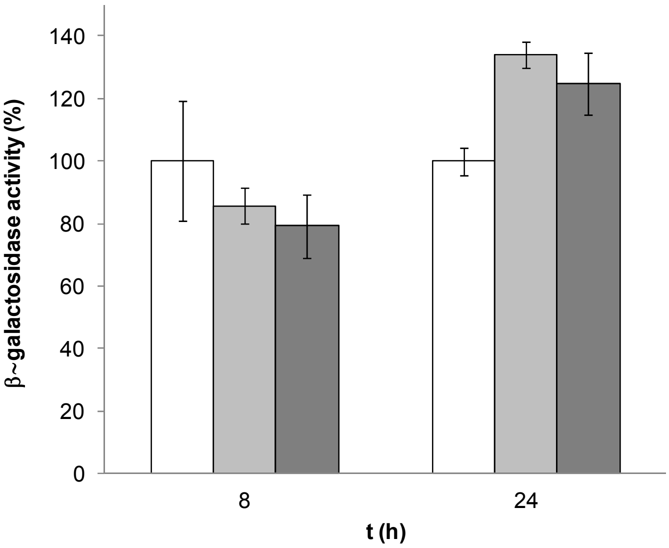

Supplementary Figure S4. **Analysis of the translational fusion Rup4959-LacZ in cfcK-64 (*argG*) and cfcK-74 (*argH*).** Relative β~galactosidase activities from the cultures harboring pMIR219 (Table S2) measured in LB supplied with Tc are plotted for the wild type (white) and mutant strains cfcK-64 (pale grey), and cfcK-74 (dark grey) at the indicated times. Average and standard deviation of two biological replicates with duplicate experimental repetitions are plotted.

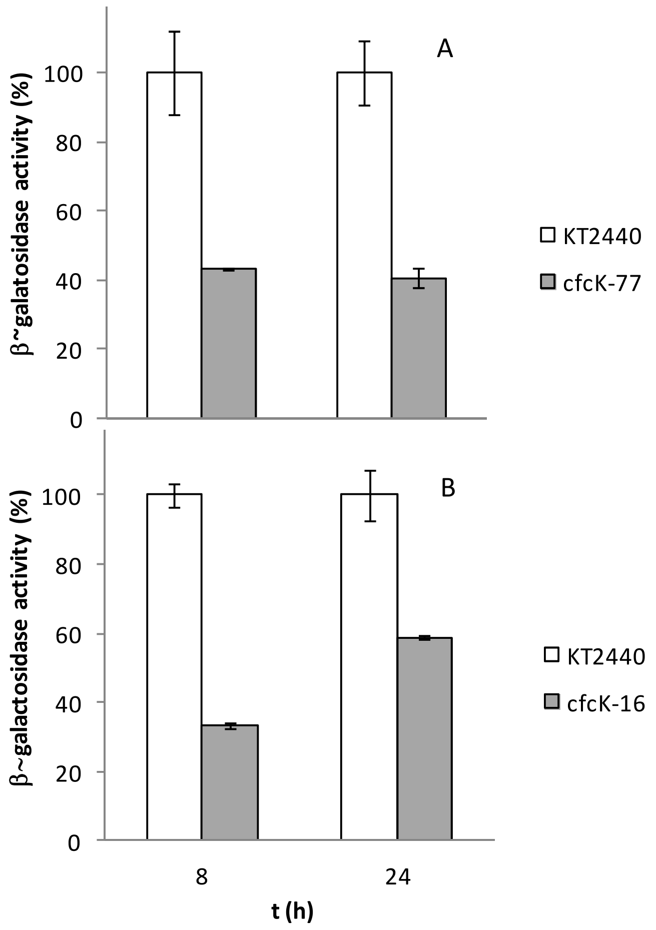

Supplementary Figure S5. **Analysis of P$_{rup4959}$::*lacZ* expression in cfcK-77 (*fleQ*) and cfcK-16.** Relative β~galactosidase activities from the cultures harboring pMIR200 (Table S2) measured in LB supplied with Tc are plotted for the wild type (white) and mutant strains (grey), cfcK-77 (A) and cfcK-16 (B) at the indicated times. Average and standard deviation of two biological replicates with duplicate experimental repetitions are plotted.

```
                                                                                           #
1DCF_A      11 LVMDEN.[2].SRMVTKGLLV.[3].CEV     TTVS     SNEECLRVV.[3].HKVVFMDVCMP.[1].VENYQIA
Rup4959     18 LVVDDY.[2].NLISMRALLA.[3].WQV     LTAS.[1].GIEALSALL.[3].VDLVLMDVQMP.[1].MDGFEVA
1DCK_B       7 HIVDDE.[2].VRKSLAFMLT.[3].FAV     KMHQ     SAEAFLAFA.[4].NGVLVTDLRMP.[1].MSGVELL
1A2O_A       7 LSVDDS.[2].MRQIMTEIIN.[4].MEM.[1].ATAP     DPLVARDLI.[4].PDVLTLDVEMP.[1].MDGLDFL
gi 714447   14 LIVDDE.[2].MRRLLEYRLS.[3].YNV     ISAS.[1].GEEALNIFR.[3].PNLIVLDIMMP.[1].LDGFGVC
gi 116853    8 LIVDDE.[2].VRRMLSTAFA.[3].FET     HCAN.[1].GRTALHLFA.[3].PDVVLMDIRMP.[1].MDGIKAL
gi 162145    9 LLVDDD.[2].IRELLETYLS.[3].FQV     RSVS     RGADFRQAL.[4].ASLAILDVMLP.[1].EDGFSLC
gi 747758   10 FAIDDS.[2].NLKILQRFLE.[3].WKV     FIAE.[1].GLTAITEIN.[3].PDIIVLDIMMP.[1].IDGFEVC
gi 105347    7 LVVEDD.[2].IRETVEEALR.[3].FEV     KSCG.[1].GADAMALLS.[6].VDLLVLDLMLP.[1].LGGLDLC
gi 744313    5 LVIDDS.[2].ERSIISDFCQ.[3].INV     TTAI.[1].GEEALEKLS.[3].PDVIILDIVLP.[1].RSGFEIC

1DCF_A      70 LRIH.[9].RPLLVALSG.[3].KSTK     EKC.[3].GLDGVLLKPVS.[ 1].DNIRDVLSDLL 127
Rup4959     78 RLMR.[6].LTPIIFLTA.[4].EAAV.[1].KGY.[2].GAVDYMFKPFD.[ 1].QILKPKVQALL 133
1DCK_B      67 RNLG.[4].NIPSIVITG.[3].VPMA     VEA.[3].GAVDFIEKPFE.[ 1].TVIIEAIERAS 119
1A2O_A      69 EKLM.[3].PMPVVMVSS.[5].SEVT     LRA.[3].GAIDFVTKPQL.[10].EMIAEKVRTAA 131
gi 714447   74 QEIR.[3].DIPIIMLTG.[2].DIVD     RIT.[4].GADDYLVKPFS.[ 1].KELEARIKSVL 125
gi 116853   68 KEMR.[4].RTPVILMTA.[4].ETAV     EAL.[2].GAFDYVIKPFD.[ 1].DELNLIVQRAL 120
gi 162145   69 RWIR.[6].CMPIIMLTA.[2].DEAD     RVI.[4].GADDYLGKPFS.[ 1].RELLARIKALL 123
gi 747758   70 RRLK.[6].EIPIIFLSA.[4].KSIV     KGL.[2].GAVDYIHKPFQ.[ 1].EEIITRLQLQL 124
gi 105347   70 RQLR.[4].HTPVLVISA.[2].SETD     RVL.[4].GADDYLVKPFG.[ 1].RELVARCRALL 122
gi 744313   65 RELK.[6].SIPIILCST.[2].TDMD     KFW.[4].GADAYITKPID.[ 1].EEFNTVIKQFI 119
```

Supplementary Figure S6. Sequence seed alignment used to determine the conserved domain cd00156 including the Rec domain of Rup4959. Marked in yellow and with # is the posttranslational modification site of phosphorylation as predicted at the Conserved Domain Database (Marchler-Bauer et al., 2015). Numbers in green indicate residue number in the amino acid sequences.

```
                                   #
1JOY_A         9 .[3].RTLLMAGVSHDLRTPLTRIRLATEMMS.[ 3].GYLAESINKDIEECNAIIEQFIDYLX 67
PP_3761      484 .[3].KSEFLANMSHELRTPLNSSLILAKLLA.[11].VKFAESIYSAGNDLLNLINDILDIAK 550
gi 6226671   878 .[3].VDKELSYVKEELKKPLEGLAFTRTVLE.[ 8].RQLIKTNAWCERQLRKILEDDLNNIE 941
gi 20091095  334 .[3].MSEFLATMSHELRTPLTAIIGFSELML.[11].RKFLGHISNSGKHLLSLINSVIDLSR 400
gi 15641456  209 .[3].KSNFLAMMSHELRTPLNAVLGLIDILR.[ 7].VELLEQMENSAELLLIIINDILDLSR 271
gi 1679757   108 .[3].KSRFLSNMSHEIRTPLIGISGMVSFLQ.[ 8].RDYTNTIQTSANSLLMIINDILDLSK 171
gi 15596833  648 .[3].RSALLASVSHDLRTPLTAMRGSIDSLL.[11].RELLESTRDEAERLDRYIQNLLDMTR 714
gi 18076415  168 .[3].EKQFVSDASHELKTPIAAIRANVQVLE.[ 5].NRYLDHVVSETKRMEFLIEDLLNLSR 228
gi 16124905  408 .[3].RSAFLANMSHELRTPLTAVIGFAALVE.[ 9].RDYVGRISTAGKALLSVINDVLEMSK 472
gi 17228687  250 .[3].EREFVSNVSHELRTPLTIVHGYLQSVL.[10].QEALEIAASEAERTIRLLQDLLDLAR 315
```

Supplementary Figure S7. **Sequence seed alignment used to determine the conserved domain cd00082 including the HisKA domain of PP_3761.** Marked in yellow and with # is the posttranslational modification site of autophosphorylation for activation of the histidine kinase as predicted at the Conserved Domain Database (Marchler-Bauer et al., 2015). Numbers in green indicate residue number in the amino acid sequences.

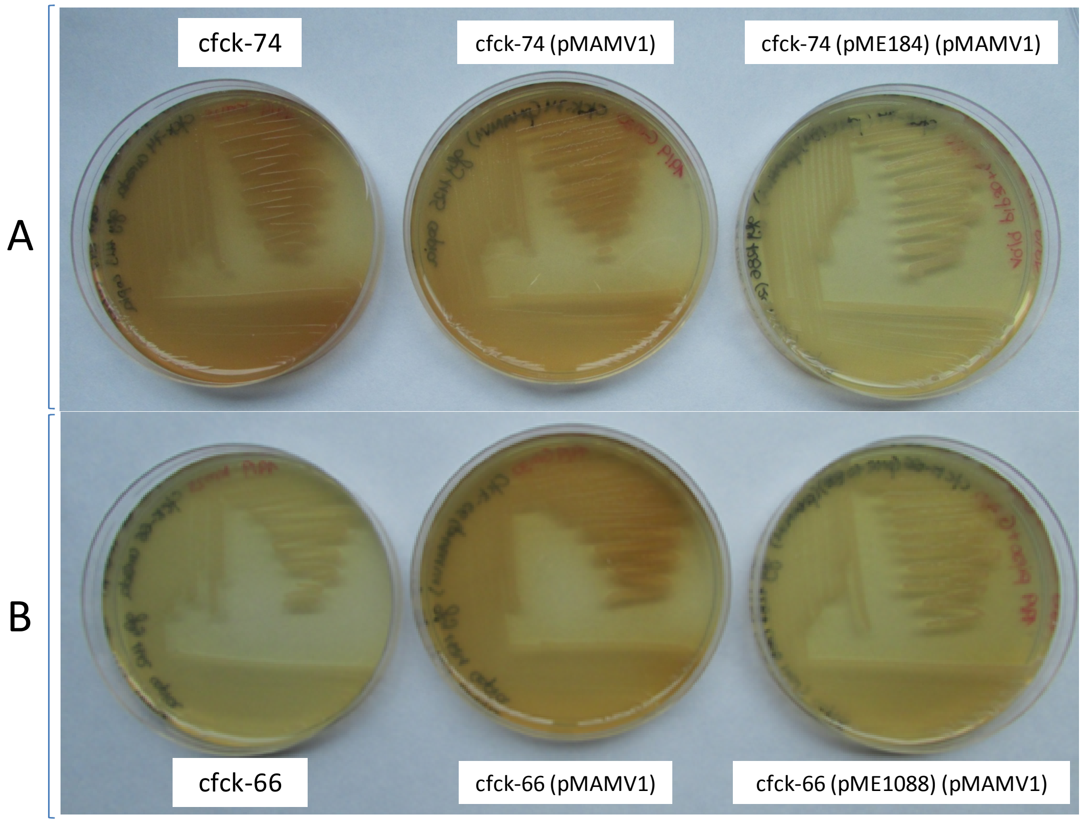

Supplementary Figure S8. Color development of mutant strains in the arginine biosynthetic pathway. (A) Mutant cfcK-74 defective in *argH* and its derivatives overexpressing the diguanylate cyclase Rup4959 from pMAMV1 or this plasmid plus an intact *argH* allele in pME184. (B) Mutant cfcK-66 defective in *argG* and its derivatives harboring pMAMV1 or this plasmid plus an intact *argG* allele via pME1088. Pictures of LB-agar plates were taken after 72 h incubation at 30°C.

A

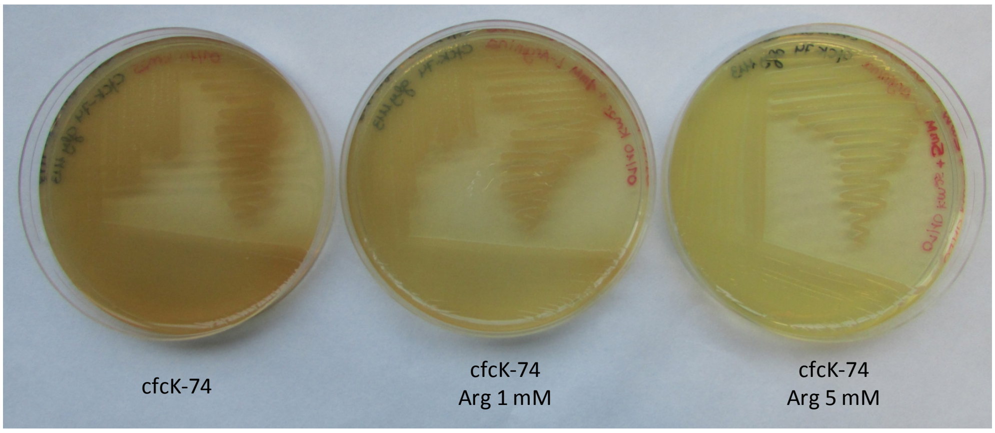

B

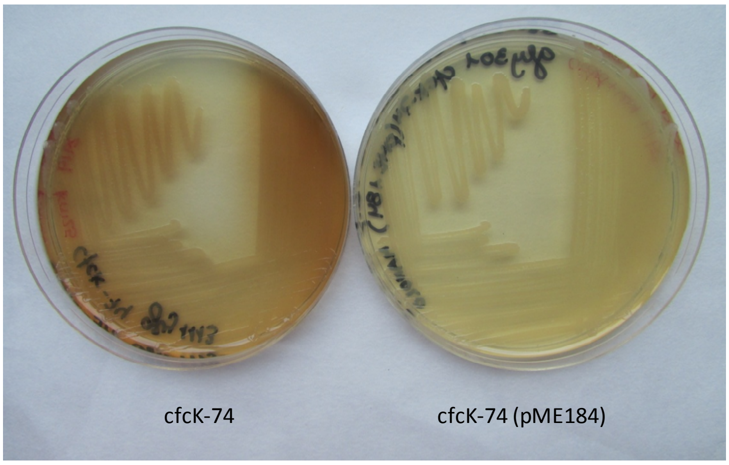

Supplementary Figure S9. Prevention of colour development in the *argH* mutant. (A) LB media was supplied with increasing concentrations of arginine. (B) An intact *argH* allele was incorporated into the strain. Pictures were taken after 72 hour incubation at 30 ºC.

Supplementary Table S1. Primers used in this work

Primers used to amplify probes

AlgUFW	AGGCGAGCTTTCGATCTG
AlgURV	AGCTTCACGACTCATGGC
FleQFW	CGCCAAGCTACAGCCAAT
FleQRV	GGCTGTCGACCATCTGCT
GacAFW	GTCGGCACTGAAACTGGC
GacARV	TGCGTCAACCATACCGTG
Km1-fw	GAGCCATATTCAACGGGAAACG
Km1-rev	CTCACCGAGGCAGTTCCATAG
PP3761FW	GCATGGTCGAGGACAACC
PP3761RV	GCACCCGTGACTGCTCTT
TcFwd	CAACCCAGTCAGCTCCTTCC
TcRev	AGCGATCCTTGAAGCTGTCC

Primers used for ectopic gene expression

ALGU2FW	gcATGCTAACCCAGGAAG
ALGU2RV	gaatTCAGGTTTCCTGCAAC
ArgG-F	AAGCTTATGGCGGACGTAAAAAAGGTC
ArgG-R	CTCGAGTCAGAGCAGCGAACGGC
ArgH-F	AAGCTTATGAGCACCGAGAAGACCAATC
ArgH-R	CTCGAGTTAGCGAGACGCCAACAGG
GacAFEco	ACTGAATTCATGATTAGGGTCTTAGTGG
GacARSal	ACTCGTCGACCGCTGCAGGTCGCCA

Primers used in Arbitrary PCR/sequencing

ARB1G	GGCCACGCGTCGACTAGTACNNNNNNNNNNGATAT
ARB6G	GGCCACGCGTCGACTAGTACNNNNNNNNNNACGCC
ARB2G	GGCCACGCGTCGACTAGTAC
ARB1-1-6	GGCCACGCGTCGACTAGTACNNNNNNGATAT
TNEXT	TGATGAATGTTCCGTTGCGCTGCC
TNINT	GACCTGCAGGCATGCAAGCTTCGGC
TNOEND	CCGCACTTGTGTATAAGAGT
TN5IEND	AGGCGGCCAGATCTGATCAA
Tnint1end	CCAGATCTGATCAAGAGACAG
TnTcO	CGCACTTGTGTATAAGAGTCAG

Suplementary Table S2. Plasmids used in this work

Plasmids	Relevant characteristics[a]	Reference or source
pMIR200	Tc^R, derivative of pMP220 with $P_{rup4959}$:: *'lacZ* transcriptional fusion	Ramos-González, M.I.
pMIR219	Tc^R, derivative of pMP220-BamHI with Rup4959-LacZ translational fusion	Ramos-González, M. I

Tc, Tetracycline

References

1. Matilla, M.A., Travieso, M.L., Ramos, J.L., and Ramos-González, M.I. (2011). Cyclic diguanylate turnover mediated by the sole GGDEF/EAL response regulator in *Pseudomonas putida*: its role in the rhizosphere and an analysis of its target processes. *Environ. Microbiol.* 13, 1745-1766. doi: 10.1111/j.1462-2920.2011.02499.x
2. Spaink, H.P., Okker, R.J.H., Wijffelman, C.A., Pees, E., and Lugtenberg, B.J.J. (1987). Promoters in the nodulation region of the *Rhizobium leguminosarum* Sym plasmid pRL1JI. *Plant. Mol. Biol.* 9, 27–39.
3. Gamper, M., Zimmermann, A., and Haas, D. (1991). Positive FNR-like control of anaerobic arginine degradation and nitrate respiration in *Pseudomonas aeruginosa. J. Bacteriol.* 173, 4742-4750.
4. Marchler-Bauer, A., Derbyshire, M.K., Gonzales, N.R., Lu, S., Chitsaz, F., Geer, L.Y., et al. (2015). CDD: NCBI's conserved domain database. *Nucleic Acids Res.* 43, D222-226. doi: 10.1093/nar/gku1221
